# Oscillatory Protein Expression Dynamics Endows Stem Cells with Robust Differentiation Potential

**DOI:** 10.1371/journal.pone.0027232

**Published:** 2011-11-03

**Authors:** Narito Suzuki, Chikara Furusawa, Kunihiko Kaneko

**Affiliations:** 1 Department of Basic Science and Research Center for Complex Systems Biology, University of Tokyo, Meguro-ku, Tokyo, Japan; 2 Department of Bioinformatic Engineering, Graduate School of Information Science and Technology, Osaka University, Suita, Osaka, Japan; 3 Laboratory for Multiscale Biosystem Dynamics, Quantitative Biology Center, RIKEN, Suita, Osaka, Japan; Centro Cardiologico Monzino, Italy

## Abstract

The lack of understanding of stem cell differentiation and proliferation is a fundamental problem in developmental biology. Although gene regulatory networks (GRNs) for stem cell differentiation have been partially identified, the nature of differentiation dynamics and their regulation leading to robust development remain unclear. Herein, using a dynamical system modeling cell approach, we performed simulations of the developmental process using all possible GRNs with a few genes, and screened GRNs that could generate cell type diversity through cell-cell interactions. We found that model stem cells that both proliferated and differentiated always exhibited oscillatory expression dynamics, and the differentiation frequency of such stem cells was regulated, resulting in a robust number distribution. Moreover, we uncovered the common regulatory motifs for stem cell differentiation, in which a combination of regulatory motifs that generated oscillatory expression dynamics and stabilized distinct cellular states played an essential role. These findings may explain the recently observed heterogeneity and dynamic equilibrium in cellular states of stem cells, and can be used to predict regulatory networks responsible for differentiation in stem cell systems.

## Introduction

Differentiation of multipotent stem cells to lineage-specific cells is one of the remarkable phenomena in developmental biology. Stem cells are defined as cells with the potential to both proliferate and differentiate into other cell types [1]–[4]. This ability of cells (or their “stemness”) is remarkable, since the cellular state must satisfy 2 conflicting properties: stability for proliferation and plasticity for differentiation. Such stemness is successively lost as the process of cell differentiation progresses during development. During this process, each cell type is robust to noise and maintains a certain protein expression pattern. In addition to this type of robustness, the course of differentiation, i.e., the timing at which cell differentiation progresses, is also rather robust while the proportion regulation in the number of cell types is achieved, i.e., the number ratio of each cell type falls within a certain range after development [5]–[7].

More than a half century ago, Waddington proposed the epigenetic landscape metaphor, in which the robustness of differentiated cell types is represented as attraction to each valley branched from the upstream [8]. In other words, cells are initially located at a shallow valley in the upstream area of a landscape, and throughout development, they fall onto one of the branched valleys in the downstream area. This proposal provided an eloquent metaphorical picture of differentiating cell robustness, and was later mathematically expressed as dynamical systems of gene/protein expression levels. In the mathematical model, each cellular state is given by a set of gene/protein expressions, which is influenced mutually through activation and repression processes. Thus, the temporal evolution of each state is represented by a set of rate equations on the various gene/protein expressions. With time, the set of expressions reaches and stays within a certain range, and this state is an attractor in the term of the dynamical systems theory [9]. If there are several attractors in the expression dynamics, each of them is set to correspond to a different cell type. From this viewpoint, the differentiation process can be described as the transition between the attractors. Indeed, this dynamical systems representation for cell differentiation was previously put forward by Goodwin [10], Kauffman [11], [12], and others. More recently, the existence of attractors has been examined experimentally using specific gene expression dynamics governed by a GRN [13]–[14].

Although the attractor picture of distinct cell types is important, it is still insufficient to understand such robustness and loss of differentiation potential. We summarize the remaining questions that should be addressed here: (1) How are the 2 conflicting functions in stem cells, i.e., proliferation and differentiation, supported by gene/protein expression dynamics and characterized by dynamical systems theory? Which characteristics of attractors distinguish between multipotent and differentiated cellular states? (2) How is the irreversible loss of differentiation potential through the course of development characterized by expression dynamics and described in terms of high-dimensional phase-space dynamics? (3) How are the course of development (timing of cell differentiation) and the number distribution of each cell type robust, regulated by cell-cell interactions? (4) What characteristics of gene regulatory networks (GRNs) are necessary for maintaining cell stemness? To address these questions, the relationship between intracellular expression dynamics and the differentiation behavior of stem cells should be further investigated.

We previously studied a class of models with intracellular reaction dynamics and cell-cell interaction [15]–[17]. Although the studies provided thought-provoking examples of the interaction-based cell differentiation process, and possible dynamical-systems concept for stemness was proposed [18], it remains still open if simpler expression dynamics consisting of just a few genes provides a model for stem-cell differentiation, and whether one can answer the above four questions from the analysis of the model. In particular, the relationship between the mechanisms of dynamic differentiation and the topologies of the intracellular reaction networks remained largely unknown.

In this study, we considered a model cell whose protein expressions are regulated by GRN with 5 genes. We report the results of extensive simulations of such cells under cell-cell interaction. We examined the dynamics of more than a hundred million GRNs by also including cell-cell interactions mediated by diffusion of proteins. We selected those GRNs that revealed differentiation through successive cell divisions. From the selected networks, we found that modeled stem cells that can undergo both proliferation and differentiation always exhibited oscillatory expression dynamics whose synchrony among cells was lost. Differentiation of the cells progressed with increasing cell number under cell-cell interactions, whereas protein expressions in differentiated cells were fixed either at a high or low level, and the cells lost potential for further differentiation and only proliferated. From the analysis of gene/protein expression dynamics, we elucidated how the cell's stemness is lost, and how robustness in the developmental course and the number distribution of cell types emerges as a natural consequence of this interaction-based differentiation mechanism. Furthermore, we unveiled network motifs allowing of stem cells. By combining these network motifs, we also succeeded in designing GRNs with hierarchical differentiation to several cell types. Some of our results are consistent with recent experimental data for embryonic stem (ES) cells, and we discuss possible candidates of feedback network structures and transcription factors that may fit our theory, while our proposed predictions should be further tested experimentally for validation.

## Results

### Model setup

We studied protein expression dynamics represented by the change in the concentration of proteins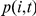, for 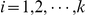 at time *t*. The expression of these *k* genes mutually influenced activation and inhibition. By starting from a single cell with given expression dynamics, it was divided into 2 cells having almost the same protein concentrations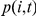 at a certain time. These cells interacted with each other mediated by diffusion of some protein through the media, where we assumed only one of the *k* proteins is diffusive. Hence, the change in protein concentration consisted of intracellular gene/protein expression dynamics and intercellular diffusion (if it is a diffusible one across cells). As the cells further divided, each cell interacted with all other cells. The Methods section contains further details of the model. Following the increase in cell number, and consequently a change in cell-cell interactions, we computed the time-course of 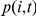 over cells. Subsequently, to check if the cells differentiated into distinct types, we computed the average expression 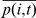 (where 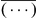 indicates the temporal average) and examined if different cells revealed distinct values. Of note, when the concentration 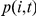 oscillates over time, the oscillations sometimes lose synchrony, and the phases of oscillations are scattered over cells, and thus 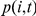 varies in cells at each time point. However, this does not indicate differentiation, because the concentrations averaged over time do not differ among the cells. Instead, the type of cell differentiation we were interested in observing had to involve differences in the average chemical compositions.

### Classification of differentiation processes

We first performed preliminary simulations of the model with a variety of GRNs, and examined if the cells indeed differentiated as their number increased, by using a certain set of parameter values. With the preliminary simulations on 5 genes with only one diffusible protein, we found several examples in which cells indeed differentiated as the number increased to 32. As for the path number, we encountered examples that show differentiation if the number is 10, whereas such examples were rare if the number is smaller. Following these preliminary studies, we ran simulations of all GRNs with 5 genes and 10 paths (a total of 145,269,760 GRNs after removing degeneracy by symmetry while including all the possibilities on the choice of diffusible protein; see the Methods for further details). This choice of networks of 5-gene, 10-path, and 1-diffusible protein is not the necessity, but computationally feasible to cover the full search, while, as will be demonstrated below, they include statistically sufficient examples that show differentiation from a cell having stemness. Here we did not exclude such networks that some nodes (genes) are disconnected from others. These include networks in which 3-gene networks are disconnected or just drove the other two genes. Indeed, the above full search of all 5-gene 10-path networks covers all possible 3-gene networks. Hence our search is also useful to obtain simple network structures that show stemness. We subsequently filtered out those networks in which cells took multiple distinct states when their number was 32 (see Methods).

**Figure 1:**
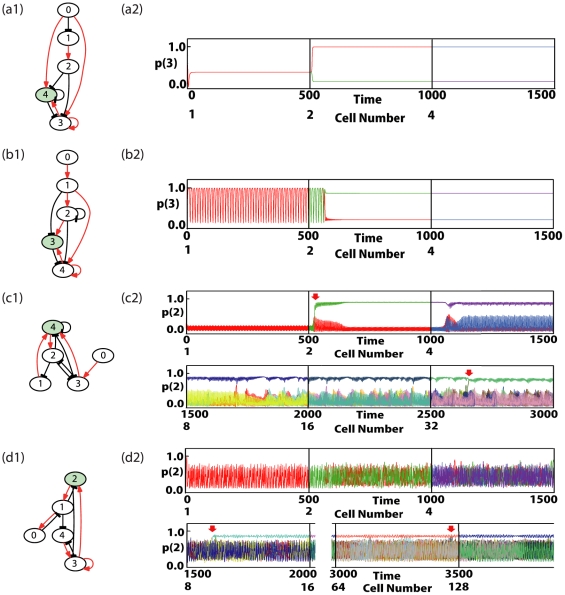
Examples of the network structure and gene expression dynamics of cells that underwent differentiation. (a1)−(d1) Gene regulatory networks. The black arrows with flat-heads and the red arrows indicate repression and activation of gene expression, respectively. The diffusible protein is represented as the green node. In (a1) and (b1), the genes 0,1,2 influence on 3 and 4, but the latter genes do not influence 0,1,2. The expressions of 0,1,2 give just a constant inputs to 3 and 4. Hence these networks are reduced to just a 2-gene system consisting only of 3 and 4. Similarly the network in (c1), the gene 0 gives a just constant input, and the network is reduced to a 4-gene system without it. (a2)−(d2) The overlaid time-series of the expression of a given protein is plotted for all cells (each with a different color). At every 500 time-steps (indicated by vertical lines), the cells divided. The colors correspond to different cells. In (a2) and (b2), after differentiation, half of cells take one identical value of concentration, and the other half take another identical value. Since the concentrations of all cells are overlaid, we could see only one color line for each type in this case. (a1, a2) Case with the Turing mechanism including differentiation from a fixed point to 2 fixed points. The protein concentration 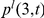 is plotted for all cells *l* (each represented with a different color). (b1, b2) Turing mechanism including oscillatory expression of 2 fixed expression levels. The protein concentration 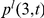 is plotted. (c1, c2) Multiple differentiations from stem-type cells via desynchronization in oscillation and switching behavior. After the first division at around 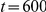, differentiation occurred. Later at around 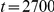 (when there were 16 cells), another differentiation from the stem-type cells occurred. The protein concentration 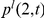 is plotted for all cells (each represented with a different color). (d1, d2) Multiple differentiations from stem-type cells with irregular (chaotic) oscillation. After the first divisions of cells, the oscillations were not synchronized over cells, and their concentrations were scattered at each time. At around 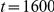, the first differentiation occurred, and another differentiation occurred again around 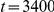 (when there were 64 cells). The protein concentration 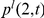 is plotted.

In summary, we found a total of 15,145 networks that showed differentiation. The most frequent behaviors of these networks belong to the Turing type, which is described below. (See Fig. 1 for typical examples of the time-series of the differentiation course.)

#### (1) Turing type (Fig. 1(a) and (b))

The initial cell state that existed in the presence of a single cell was destabilized upon the cell-cell interaction that occurred when the cells divided into two. The cellular state of the single cell was unstable upon cell-cell interaction, and it never reappeared after the cell number increased. This differentiation is understood straightforwardly upon consideration of the classic mechanism for the Turing pattern [19] (also see [20]). A common network was observed in these examples, as shown in Fig. 1(a). In these examples, there existed a pair of proteins that satisfied the following relationship: one of the proteins (termed as the activator) activated the expression of itself and the other protein, while the other protein (termed as the inhibitor) repressed the expression of itself and the activator. The inhibitor protein was diffusive. Now consider 2 divided cells whose concentrations of these proteins slightly differed. The cell with a higher (lower) concentration in the activator further increased (decreased) its concentration, according to the expression dynamics. This increase (decrease) was compensated by the increase (decrease) in the inhibitor, if there was no cell-cell interaction (i.e., there was no diffusion of the inhibitor over cells). However, in the presence of the diffusion, the inhibitor concentrations of the 2 cells were equalized, which prevented the compensation from occurring. Subsequently, the cell with a higher concentration of activator further increased its concentration, and vice versa. Thus, the difference in the activator concentrations between the 2 cells was large enough to reach 2 distinct states, and the concentrations were subsequently stabilized. Indeed, this mechanism and the network structure are those proposed as the Turing pattern.

This activator-inhibitor network structure is simple, such that a large number of networks had this structure in its core portion. Among the networks of 5 genes and 10 paths, we found 3794 networks that belonged to this class.

Furthermore, this Turing mechanism could be combined with oscillatory dynamics. In a network that produced oscillation in protein expression as a single cell, when the cell divided into 2 cells, one of the resultant cells had a fixed higher concentration of the activator protein, whereas the other cell had a lower concentration (see Fig. 1(b)). A total of 8875 networks exhibited this behavior.

However, these examples, albeit commonly observed, did not satisfy the postulate for a stem cell, which must be able to both proliferate and differentiate. Indeed, the initial cell type appeared only at an initial stage with only 1 (or a few) cell(s). Such a cell type differentiated into 2 types after the division and subsequently disappeared. In contrast, the 2 cell types that appeared only proliferated, but failed to differentiate. Hence, at any stage, there was no cell type that could both proliferate and differentiate.

**Figure 2:**
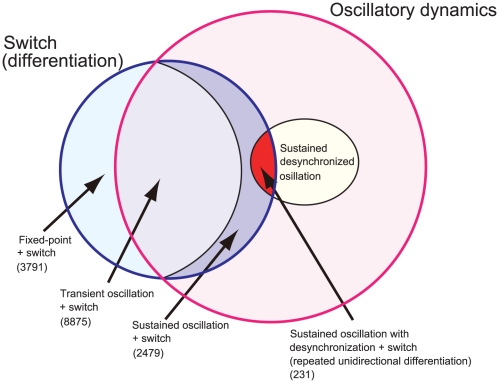
Venn diagram on the classification of GRNs. The blue circle is a group in which a Turing-type switch mechanism occurred by cell-cell interaction, whereas the pink circle indicated the group of GRNs in which protein concentrations showed temporal oscillation. The former group showed at least one differentiation event. The oscillation group is then classified into with and without sustained oscillations, and the latter is classified by whether the oscillations remain desynchronized over cells. The GRNs that showed repetitive differentiation, i.e., having cells with stemness, were given by the intersection of the group with sustained desynchronized oscillations and the group possessing Turing-type switch mechanism (displayed by red color).

There remained 2476 networks in which the oscillation remained after cell division. In fact, among such networks, there were examples that satisfy the requisite for stem cells. (See Fig. 2 for the Venn diagram on the classifications of GRNs with regards to the differentiation and oscillation).

#### (2) Differentiation through sustained oscillatory dynamics (Fig. 1(c) and (d))

To select such networks in which cells can both proliferate and differentiate, we further filtered such networks that revealed plural differentiations in the same direction repeatedly throughout cell division to up to 256 cells. Here, by the restriction on this ‘unidirectional’ differentiation, we discarded the cases with type 1 → type 2 and type 2 → type 1 back again, and included only the cases with repetition of type 1 → type 2 (see Methods). Upon this selection, we identified 231 networks. (This list of networks appears in Fig. S1.) These networks provided a system in which differentiation from the stem cell type existed even after cell divisions.

For these 231 networks, oscillatory protein expression existed at the initial stage. As the cell number increased, the synchrony of the oscillations of the cells was lost. The phases of oscillation were scattered across the cells. (This is understood in the context of the theory of the coupled dynamical system [21].) Upon the cell-cell interactions, some expression levels were up-regulated and down-regulated aperiodically, as shown in the lower group in Fig. 1(c2) for *t>1200,* and in Fig. 1(d2). Expression of some proteins reached a high level temporarily but was subsequently suppressed back to a low level. Now, differences in expression levels between cells were no longer restricted to the phase of oscillations, but the absolute expression levels, i.e., time-averaged concentrations were differentiated and this difference was amplified. Some cells deviated from the original attractor (see the events at *t*∼*2650* in Fig. 1(c2) and those at *t* ∼*1600* and ∼*3450* in Fig. 1(d2)).

**Figure 3:**
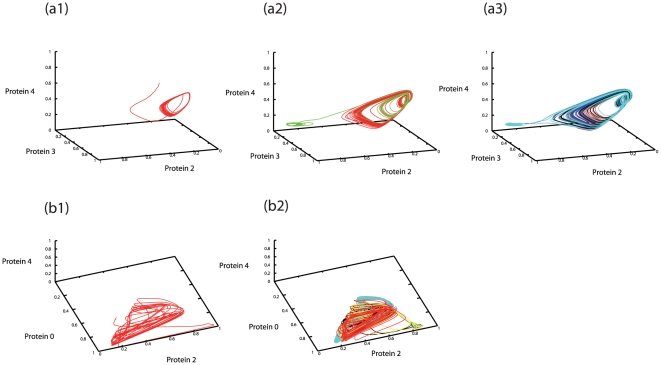
State differentiation represented by orbits in the state-space of 3 protein concentrations. (a) The time evolution of 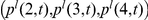 is plotted, for the gene regulatory network (GRN) shown in Fig. 1(c). Plotted over *0<t<100* (a1; single cell), *500<t<600* (a2; 2 cells 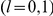 with different colors), and *2600<t<2700* (a3; 6 from 32 cells 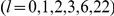 with different colors). Differentiation from the original attractor (right part) to a new state (left) progressed. (b) The time evolution of 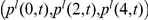 for the GRN in Fig. 1(d). Plotted over *0<t<100* (b1, single cell), *3400<t<3500* (b2; 8 from 64 cells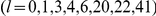 plotted with different colors). Differentiation from the original attractor with aperiodic oscillation (left) to a new state (right) progressed.

This process of differentiation, plotted in the state space {*p(i)*}, is displayed in Fig. 3, as well as in Movie S1. With the cell-cell interactions due to diffusion of a protein over cells, in addition to the positive feedback process within the GRN, the switched states were stabilized without returning to the original oscillation again. For such cells, the oscillation amplitude was drastically reduced. By comparing two types of cells after they reached steady behaviors (*t>1600*) in Fig. 1(c2) and (d2), the amplitude of the oscillation of the differentiated type was much smaller (about 1/10 or less) than the other (see also [15], [22], [23]). Their expression pattern was fixed and maintained. Hence, differentiation from cells with oscillatory dynamics to cells with fixed expression states progressed. The oscillation of the other, original cell type was sustained by keeping a large amplitude as shown in Fig. 1(c2) and (d2).

This differentiation was unidirectional, and only transitions from cells with the original type to the other cell type with a fixed expression state occurred. Cells with the larger-amplitude oscillation either proliferated or differentiated after their division. Hence, this case met the postulate for a stem cell. In contrast, the other cell type with a fixed state proliferated only, implying the loss of differentiation potential.

At the event of differentiation, oscillations were desynchronized over cells, and the oscillation pattern was not periodic but irregular (as known as chaotic dynamics). For most networks we examined here, such irregular (aperiodic) oscillation did not exist as single-cell dynamics, but instead appeared only after cell division occurred and oscillation lost synchrony as a result of cell-cell interactions. However, for a few networks, irregular oscillation in protein expression dynamics existed for the initial cell type as an attractor of a single cell (see Fig. 1(d)).

The classification of GRNs is summarized as follows (see Fig. 2): First, there are two conditions, one for (I) the switch by Turing-type mechanism due to cell-cell interaction (blue circle) and the other for (II) oscillatory expression dynamics (pink circle). The former case showed at least one differentiation event. The GRNs that belong to only the former case (I) corresponded to the case of Fig. 1(a). Then, we focused on GRNs that satisfy both the conditions (intersection of I and II). This group is classified by whether the oscillation was sustained or not. If not, such network corresponded to the case of Fig. 1(b). Now, the group of sustained oscillation is classified if the oscillations remained desynchronized over cells or not, i.e., whether the phases of oscillations were scattered over cells or synchronized. If synchronized, such GRN lost the potentiality for differentiation after a differentiation event, and could not show repeated differentiations. Finally, those GRNs with sustained desynchronized oscillation with the Turing-type switch showed repeated differentiations, from a cell type preserving the potentiality both for proliferation and differentiation.

Thus the condition for expression dynamics to have repetitive differentiations is given as follows: (1) Oscillations were sustained after cell divisions; (2) Desynchronization of oscillations over cells was sustained; (3) Due to the cell-cell interaction, Turing-type mechanism worked to suppress the oscillation amplitude for some cells; By (1) and (2), the potentiality for differentiation was preserved. By (3), the cells differentiated into two types, one sustaining a large-amplitude oscillation, the other with only tiny oscillation or without it. The original cell type preserved desynchronized large-amplitude oscillation, and the differentiation occurred repeatedly from it with the increase in cell number.

Of note, sustained oscillation with desynchronization was necessary but not sufficient. Indeed, we studied GRNs in which aperiodic (chaotic) oscillation in protein expression (which amplifies differences in cells) existed. Then, phases of oscillation were scattered over cells, but the expression levels were identical among cells in terms of the temporal averages. Thus, differentiation in composition of proteins did not necessarily follow (see Fig. S2 for such an example).

### Dynamical systems mechanism

The differentiation process described above can be understood as a change in the flow structure in dynamical systems due to the change in the interaction term (see Fig. S3(a)). Up to some cell number, the initial oscillation state was an attractor, even upon interaction with other cells of the same type. As the cell number increased, the interaction term generated another stable state. With the interaction term, the orbit crossed over the basin of attraction to itself and was forced to another state, resulting in differentiation to a novel state. Subsequently, under a sufficient number of differentiated cells, the interaction term strengthened the attraction to the initial oscillatory state, such that the orbit no longer crossed the barrier of the basin of attraction to itself, which ultimately led to proliferation (see Fig. S3(a)). When the number of cells from the original oscillatory attractor further increased, it generated a flow to the outside of the basin of attraction, resulting in a switch to other states. In this manner, both proliferation and differentiation coexisted in the interacting cells.

Now, the cell “stemness” was represented by the sustained desynchronized oscillation in the expression dynamics. As long as the original large-amplitude oscillation with instability was sustained, the differentiation potential remained under the presence of Turing-type switch mechanism. Hence, with the further increase in the existing cell number, stem cell differentiation continued. In some cases, such oscillation was lost after some cell divisions, and consequently differentiation no longer occurred.

As for the differentiated state that lost such cell stemness, there were 2 classes. In most cases (188 networks), such a state did not involve an attractor as a single-cell state. When isolated as a single cell, the state was no longer stable. Moreover, it was stabilized only by cell-cell interaction. However, for the remaining 43 networks among the 231 networks we screened, the differentiated state also involved an attractor with single-cell dynamics. For such networks, the single-cell expression dynamics had 2 attractors: 1 for the stem cell with sustained oscillation that had a large basin of attraction, and the other for the differentiated state that lost such oscillation. For most initial conditions, the stem-cell attractor was reached, whereas the other “differentiated” attractor had a particularly small basin of attraction and was not easily reached with single-cell dynamics. When starting from a cell with the oscillation attractor and as the cell number increased beyond some value, cells could not remain at the original oscillation attractor due to the cell-cell interaction. Subsequently, some cells differentiated to the other cell type, which remained stable.

### Robustness in the cell number ratio

**Figure 4:**
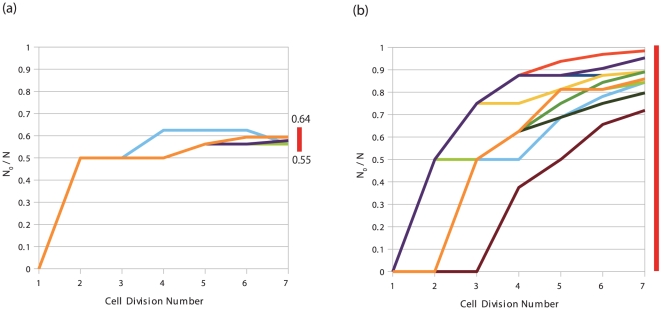
Ratio of the number of the differentiated cells to the total cell number, 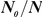. The horizontal axis 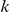 denotes the cell division event, and the total number of cells 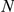 is given by 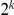. The temporal course of the fraction is plotted over 10 runs, with each represented by a different color, upon inclusion of a large amount of noise 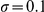 at each division. The bar at the right end indicates the range of the fraction that stably exists, which was computed at 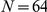 by changing the initial number of 1 cell type. (a) Plot for the GRN in Fig. 1(c). (b) Plot for a network that originally produced 2 attractors that were selected by the simulation with a large amount of noise (see the Discussion section). In this case, any cell type number distribution was allowed.

The change in flow structure described above naturally results in the regulation of the differentiation frequency in forming stem cells depending on the number and states of the surrounding cells, in addition to the robustness in the cell type number distribution [16], [24]. To investigate this point, we carried out simulations of the present model with a fixed total cell number at *N* (instead of the simulation starting from a single cell), and set the initial cell numbers of stem and differentiated cells to *N_0_* and *N−N_0_*, respectively. By varying *N_0_*, we examined whether each cellular state was stable in the presence of cell-cell interactions at each cell type distribution (see also Fig. S3(b)). In the examples we studied here, there was a certain range of *N_0_* at which both of the 2 types coexisted stably. This range is indicated as the bar at the right side of Fig. 4(a), for the GRN of Fig. 1(c). For example, the states *N_0_ = 0* and *N_0_ = N* were unstable, and the stem cells with oscillatory dynamics coexisted with the differentiated cells only within the range *0 <N_l_* ≤ *N_0_* ≤ *N_u_<N*, where the upper and lower limits, *N_u_* and *N_l_,* were dependent on the network and parameters of the protein expression dynamics. This range [*N_u_*, *N_l_*], as shown in Fig. 4(a) was much smaller than [*0,N*]. This result implies that the cell number regulation works in the model. When started from a single cell, the number ratio of each cell type fell on a much narrower range than the above range *N_l_* ≤ *N_0_* ≤ *N_u_*, even if the system was simulated under a large noise level at each cell division or during expression dynamics (see Fig. 4(a)). The timing of the differentiation from the initial cell type was also stable in each course, even though we simulated the developmental course with a large amount of noise. The developmental time-course of differentiation was rather stable. This result in Fig. 4(a) corresponded to the case in which the differentiated cell type was not a single-cell attractor. If the differentiated cell type was also an attractor of single-cell dynamics, then *N_l_ = 0*. However, for development from a single cell in most initial conditions, the number ratio after development fell within a small range (data not shown).

### Network structure: Combination of “oscillation modules” and “switch module”

**Figure 5:**
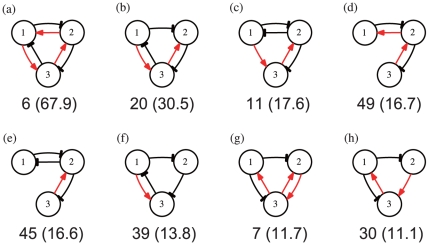
Eight frequent network motifs consisting of 3 genes. The values represent the frequencies of the motif appearances among the filtered 231 networks. The values in parentheses are the ratios of the actual appearance values to the corresponding expected values in random networks. Motifs in (a), (b), and (f) include a repressilator. i.e., a loop of 3 successive inhibitions. Motifs in (a), (b), and (d) include more than 2 positive-negative loops, i.e., 2 genes that activate and inhibit each other.

The common expression dynamics in the stem cells (i.e., oscillation maintaining their stemness) suggested common characteristics in the structure of GRNs in the stem cells. To unveil such common characteristics, we numerically examined the frequency of three-node network motifs [25] that appeared in the selected 231 networks. Ignoring the direct self-feedback for the moment, there were a total of 132 motifs with 3 nodes that had all varieties of positive and negative signs. Some network motifs appeared much more frequently in the selected 231 networks than those expected from random networks of networks with 5 genes and 10 paths. In Fig. 5, we presented 8 such three-gene network motifs whose frequency was 10 times more than that of the random networks, and was larger than 5 (to discard rare cases).

Among these 8 motifs, several types of negative feedback loops frequently appeared. The first type involved a three-gene negative feedback loop in which gene 1 repressed gene 2, gene 2 repressed gene 3, and gene 3 repressed gene 1, which is denoted by (− − −); this type of a loop with 3 successively suppressing genes is also called a repressilator (R, in short) [26]. This type existed in Fig. 5(a)(b)(f). The second type was a two-gene negative feedback loop in which gene 1 activated gene 2 and gene 2 repressed gene 1, which is denoted by (+ −); this type is also termed a positive-negative loop (pn loop). This type appeared three times in Fig. 5(a), twice in Fig. 5(b)(d), and once for the rest 5 motifs in Fig. 5. The third type was a three-gene loop in which gene 1 activated gene 2, gene 2 activated gene 3, and gene 3 repressed gene 1, which was termed as a ppn (+ + −) loop. This type existed in Fig. 5(c)(g)(h).

Furthermore, all 8 motifs included multiple negative loops. Three motifs included a combination of R and pn loop(s), and 4 included sequential pn loops (gene 1 repressed gene 2, gene 2 repressed gene 3, gene 3 activated 2, and gene 2 activated gene 1). Among the latter 4 motifs, 2 also included R and belonged to the first type of motif. The other 3 motifs included both a pn loop and a ppn (+ + −) loop. Hence, all of these three-gene motifs had plural negative feedback loops, suggesting that the combination of negative feedback loops was rather common in networks that generated stem cells.

To examine the hypothesis that a combination of negative loops is relevant to generation of stem-cell dynamics, we counted the networks that included (i) R and pn loops, (ii) plural pn loops, (iii) R and ppn loops, or (iv) pn and ppn loops. The frequencies of these types were 92, 122, 37, and 112, respectively, among the 231 networks, which were much larger than the expected numbers for the random networks, which were 16.9, 35.3, 6.3, and 46.2, respectively (*p*<10^−8^, determined by a randomization test).

By analyzing common motifs in the GRNs that generate stem cell dynamics, we found that they generally have 2 “modules” with different roles, as described below.

#### (1) Negative feedback loop for oscillation module

The fact that the GRNs of stem cells generally had plural negative loops matches well with the dynamical systems mechanism discussed in the last section. As mentioned, oscillation in protein expression was generated by a negative feedback loop. The simplest negative loop in the present model consists of the above-mentioned pn loop. The GRN of this two-gene loop alone does not generate oscillation, but adding an activating regulatory path to the loop to activate the genes, oscillation is generated. In addition, combining this negative loop successively also generated oscillatory protein expression. Another typical gene network that produced oscillation is the R. Due to its multiple negative feedback loops, aperiodic oscillation could be generated. Recall that a simple oscillation was insufficient to lead to the instability necessary for differentiation. However, the existence of multiple negative feedbacks can produce several oscillation components, which makes reaching instability easier.

#### (2) Switch module

For a switch to a differentiated state to occur, both mechanisms were necessary to amplify a tiny difference between cellular states by positive feedback and to fix the difference in cellular states. There are a variety of possibilities for introducing such positive feedback, but a simple module is provided by the Turing mechanism, in which both positive and negative self-feedback were added to the pn loop, as shown in Fig. 1, where the protein from the gene with negative self-feedback was diffusive. In fact, a combination of a series of two-gene negative feedback (+ −) loops with a positive self-feedback, as shown in Fig. 5, was frequently observed in networks that generated stem cells. This combination apparently amplified and fixed the cellular state difference.

The relevance of oscillation and the switch modules explains why the combination of negative loops was dominant. That is, they were necessary to provide oscillations. Furthermore, multiple negative loops allowed for complex oscillations that led to the instability required for differentiation. The pn loop, in conjunction with the self-feedbacks, offered a switch module. There were only 19 networks among the 231 that did not include multiple R, pn, and ppn motifs. Most of these exceptional networks included negative feedbacks of more genes, such as a four-gene loop consisting of (+ − − −) or (+ + + −). Furthermore, with the appropriate combination of self-feedbacks, both switching and oscillation modules were produced in these cases.

**Figure 6:**
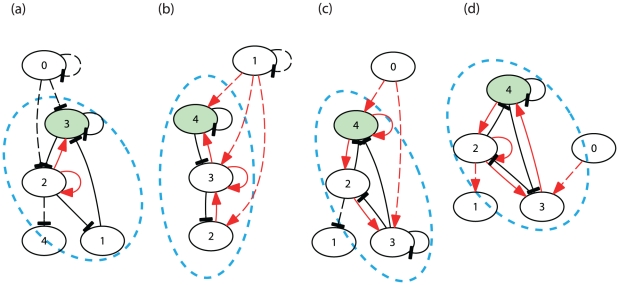
Four gene regulatory networks consisting of 3 genes detected from our extensive simulations of five-gene networks. Two of the five genes were either detached (that is ‘0’ not shown in (b)), only received input from others, or only gave input(s). In the last case, the genes only modify the threshold parameter(s) of other gene(s). The rest of the 3 genes (1, 2, and 3 for (a) and 2, 3, and 4 for (b)−(d)) that were relevant to the dynamics are enclosed by dotted ellipses. (a)(d) The network includes a repressilator, a loop of 3 successive inhibitions, and a Turing-type activator-inhibitor loop. (b)(c) The network includes 2 positive-negative loops, one of which forms a Turing-type activator-inhibitor relationship together with self-feedbacks.

Of note, the dominant three-gene motifs often consisted of a series of two-gene negative loops and a R. Adding some self-feedback to these motifs produced GRNs that generated stem-cell expression dynamics. Among all possible GRNs within the 5 genes that we inspected, we found 4 networks (Fig. 6) that could generate the dynamic differentiation process discussed above. Two networks ((a), (d)) among these 4 networks consisted of a R and Turing-type motifs. The other 2 networks ((b), (c)) consisted of a series of Turing-type motifs only. All of these networks showed sustained oscillations with instability for switching in protein expression as the cell number increased, and led to progression of the differentiation from stem cells. In particular, the expression dynamics of stem cells shown in (b) and (d) maintained irregular (chaotic) oscillations, and preserved the potentiality for differentiation even after two differentiations. (See Fig. S4 for the differentiation dynamics provided by the GRN shown in Fig. 6(d).)

Relevance of positive and negative feedback loops to robust and tunable biological oscillation was noted at a single-cell level [27], while their role to flexible biological switches was discussed by applying bifurcation analysis [28]. In our case, these feedback loops work as a generator for complex intra-cellular oscillation, and are also essential to the switch under the existence of cell-to-cell interaction.

### Designing GRNs that provide hierarchical differentiation from stem cells

Following the logic presented in the last subsection, we could indeed construct networks that provide hierarchical/multiple differentiation from stem cells merely by combining the network modules for oscillatory dynamics with both negative feedback loops and a switching module of the Turing mechanism. Thus, we further adopted a GRN module that exhibited chaotic oscillation, in which the small differences in protein concentrations between cells was amplified.

**Figure 7:**
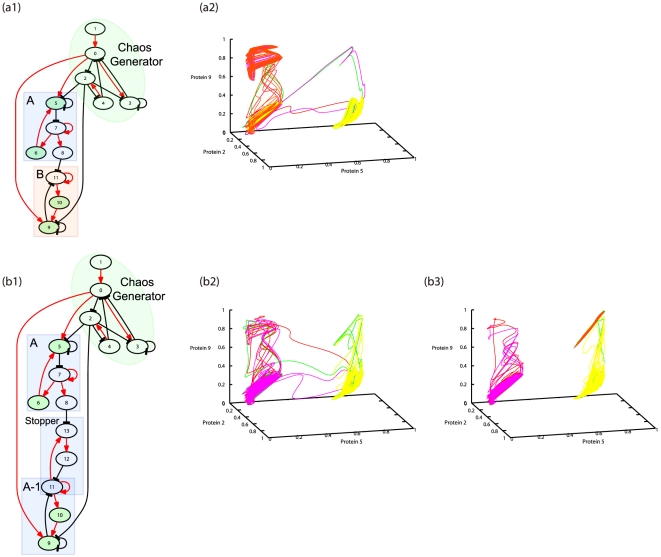
Design of the networks with multiple or hierarchical differentiations. (a1) Gene regulatory networks (GRNs) from a stem cell (S) to 2 types A and B. (a2) Differentiation of cells represented by orbits in the state-space of 3 protein concentrations. 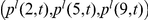 is plotted for 6 cells over 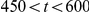. (b1) GRN to generate hierarchical differentiation from the stem cell (S) to cell type A, and subsequently to type A1. (b2) Differentiation of the stem cell (S) to the cell type A, represented by orbits in the state-space of 3 protein concentrations 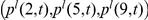 is plotted for 6 cells over 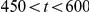. Differentiation of the original attractor (left) to a new state (right) progressed. (b3) Differentiation of A to A1 represented by the orbits 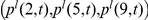 is plotted for 6 cells over 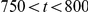. Throughout Fig. 6, 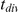 was set to 160, instead of 500, to allow observation of several differentiations within a shorter time span. The diffusion constant 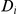 is set to 0.125.

There are several choices to be made for each module, and accordingly there are various ways to construct GRNs that generate complex differentiation from stem cells. As shown in Fig. 7(a) and (b), we choose to use an oscillation motif with 5 genes (genes 0–4) to produce aperiodic oscillation, and the Turing-type motif with 3 genes (genes 5–7) to make a switch with some delay. In fact, using a GRN that consisted of the 2 modules, we confirmed differentiation from stem cells with the increasing cell number.

Next, we combined a single oscillation module with several switch modules of the Turing type. The oscillation module was chosen to show chaotic dynamics (irregular oscillation). First, by adding switch modules in parallel (see Fig. 7(a) and Movie S2), differentiation with multiple branchings as either S→A or S→B progressed with the increasing cell number. Each differentiated cell type corresponded to an “on” state with the expression of the protein at each switch module. Here, we added a path in the GRN such that 1 module (A in Fig. 7(a1)) inhibited the expression of the other module (B). Thus, the genes in module B were expressed only if the expression of module A was suppressed, whereas the initial S state showed temporal changes between the “on” and “off” states of these expressions. The temporal variation in the expression patterns of the initial S state was large, and ranged over states close to those of types A and B.

Next, by combining the switch modules in sequence, hierarchical differentiation of?S→A/A→A_1_ was generated (see Fig. 7(b) and Movie S3). (Of note, we added a “stopper” in the network so that the genes in part A1 (genes 9–11) were not expressed by themselves, but were instead expressed only if the genes in part A were expressed. This “stopper” (genes 11 and 13) inhibited the activation of gene 11 (with some delay) unless gene 13 was suppressed by the expression of part A (gene 7 via gene 8).) Using this approach, the initial cell type had the potentiality to produce the other 2 cell types. The temporal variation in the expression pattern decreased as the differentiation of S→A→A_1_ progressed. The initial cell type showed large-scale changes in the expression pattern, covering those close to those of cell types A and A_1_. However, the type A cells showed a change limited only between the states close to those of the cell types A and A_1_, and the expression of state A_1_ was almost fixed (see Fig. 7(b2) and (b3)). The hierarchy in the differentiation of cell types corresponded to the hierarchy in the range of the phase space within which the expression of each type could vary.

Using these modules, one can generate more complex differentiation as in the hematopoietic system, with both multiple branching and hierarchical differentiation. An example of the differentiation process S→A,B/A→A_1_/A→A_2_/A_2_→A_3_/A_3_→A_4_/B→B_1_ is given in both Fig. S5 and Movie S4. In principle, complex hierarchical differentiations are designed in the same way.

## Discussion

### Comparison with an alternative view: Noise-induced differentiation

**Figure 8:**
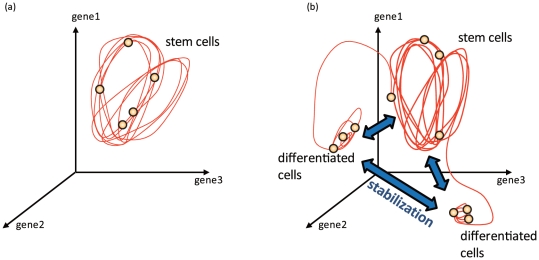
Schematic representation of our differentiation scenario from a stem cell, represented by dynamical systems of the state space of protein expressions. (a) and (b) represent before and after differentiations, respectively.

In this study, we presented the dynamic differentiation process of stem cells, in which the transition between cellular attractors is caused by dynamical instability arising from cell-cell interactions. According to our theory, expressions of some proteins oscillate in stem cells. As cells divide the synchrony of oscillations across cells are lost. With the cell-cell interaction, then, some cells no longer keep the oscillation pattern and switch to a different state which loses the oscillatory expression. The differentiated cell type(s) and the original stem cell are mutually stabilized by cell-cell interaction, as long as the number ratio of each cell type is within a certain range, and thus the ratio is regulated (see Fig. 8 for schematic representation of this scenario).

An alternative mechanism for the switch of cellular states is noise in protein expression, which has been reported previously [29]. Due to stochasticity either by molecular fluctuation or extrinsic noise, the protein concentrations of cells are modified, which may perturb expression levels to enter a basin of different attractors. When such a switch occurs, it is interpreted as cell differentiation; in fact, some experiments have shown that noise in protein expression plays an essential role in the differentiation process of *Bacillus subtilis* [30]. However, there are some drawbacks in this scenario of noise-driven differentiation. First, in noise-driven switching, any number distributions of the 2 cell types are realized. We found that the number ratio of each type of cells was highly variable in each run of the developmental courses (Fig. 4(b)). This is in strong contrast with our interaction-based differentiation, where the ratio after development was sharply determined. In the scenario we discussed, even after introducing a larger amount of noise, this ratio fell within a narrow range, as shown in Fig. 4(a). (By suitably introducing cell-cell interaction into multiple-attractor dynamics, robustness in cell-number ratio would be possible.)

Another drawback in the stochastic switching scenario is that the differentiation process itself can vary at each developmental course because the process is governed solely by noise. The timing (generation) for cell differentiation was dependent on each case (Fig. 4(b)). In contrast, in our interaction-based scenario, the differentiation consistently started when the cell number reached some value. This value was dependent on only the GRN and parameters governing the expression. That is, once the network and parameters are given, the model is quite robust against added noise during development. Thus, the differentiation course is noise-tolerant.

The third drawback of the stochastic switching scenario is the necessity to tune the noise amplitude in order to achieve irreversible differentiation from multipotent stem cells. In general, noise can introduce transitions in both directions between the 2 cell types 1 and 2. To differentiate the transition rates from type 1 to type 2, and vice versa, the stability of the 2 attractors should be substantially different. However, if the noise amplitude is large, then the 2 transition rates are quite similar, and there is no distinction between stem and differentiated cell types. By decreasing the noise level, the rates of the 2 transitions can become substantially different if the stability of the 2 attractors is substantially different. However, if the noise magnitude is too low, the transition itself can hardly occur. Thus, it is necessary to tune the noise level. Furthermore, finer tuning of the model is necessary to make hierarchical and/or plural differentiations. In contrast, the design in the last section did not require fine-tuning of the parameter values.

According to our hypothesis, sustained irregular oscillation in expression dynamics spontaneously generates the transitions from stem cells via cell-cell interactions, which also leads to the robustness of the cell type population ratios.

### Potential experimental verification of our hypothesis

According to our theory, there are basic characteristics of stem cells that either have been or can be confirmed experimentally. Some examples of these characteristics are as follows:

#### (1) Larger cell-cell variation

According to our theory, expression of some proteins exhibits a large temporal variation with a rather slow time scale, such that the concentrations of some proteins significantly differ in cells at each snapshot in time. This large variation is revealed as heterogeneity in ES cells, as demonstrated by recent advances in single-cell measurements. Such previous studies have revealed that the expression patterns of pluripotent or multipotent stem cells are heterologous, and that a dynamic interchange exists between subpopulations. For example, Toyooka et al. found that the expression level of Rex1, which is widely used as a marker of pluripotency and is strongly expressed in the inner cell mass, exhibited heterogeneous expression levels in Oct3/4-expressing ES cells, and Rex1-positive and -negative ES cell subpopulations were in a state of dynamic equilibrium [31]. Similar heterogeneity of expression levels and a dynamic equilibrium between subpopulations have also been observed for Nanog [32] and Stella [33] in ES cell populations, and for Sca-1 [14] in hematopoietic progenitor cells. These findings indicate that the transitory dynamics observed in stem cell populations reflect the course of development *in vivo*, and thus they play an essential role in the cell fate determination during developmental dynamics.

#### (2) Temporal variation in concentrations of some proteins

The source of heterogeneity in the cellular states and the driving force of transitory dynamics observed in stem cell systems still remain unclear. One possible mechanism responsible for both the heterogeneity and the transition is the noise in the expression dynamics [29]. Fluctuations in mRNA and protein numbers can drive transitions between cellular states, which may result in the observed heterogeneity and regulation of the differentiation frequency. However, the stochastic switching (noise-driven) mechanism for cellular differentiation has several drawbacks, as discussed above.

An alternative potential mechanism for the observed heterogeneity is oscillatory expression dynamics that we proposed in this study. Indeed, some recent studies support the existence of temporal changes over different expression patterns or oscillatory expression dynamics in stem cells. For example, Huang and his colleagues showed that, in hematopoietic progenitor cells, the expression dynamics exhibited transitions over quasi-stable states, which suggests the existence of slow dynamics in the GRN [14]. Furthermore, Kobayashi and Kageyama recently used single-cell real-time imaging to show temporal oscillation in the Hes1 expression level of ES cells, and found that the phase of this Hes1 oscillation controlled the differentiation fate choice toward neural and mesodermal differentiations [34]. Furthermore, the observation of oscillations in Hes1-downstream genes Dll1 and Gadd45 g suggests that the oscillations in these expression levels propagate through gene network regulation of differentiations from ES cells.

To test our hypothesis experimentally, it is crucial to further investigate the time-course of the protein expression levels at the single-cell level. By analyzing the time-series of single-cell-level expression data, one can extract information about the trajectory of cellular dynamics. For example, one can use standard time-series analysis to distinguish whether the variation of expression over time is due to stochasticity in expression dynamics only, or if it originates from the high-dimensional dynamics inherent to cells.

#### (3) Network structure

Another approach to test our hypothesis is to identify GRNs that can maintain the dynamic differentiation process of stem cells though oscillatory expression dynamics and cell-cell interactions, as we predicted. Our model simulation suggested that 2 types of network modules, i.e., an oscillation module composed of feedback loops and a switching module composed of activator-inhibitor regulations, are generally observed in the GRNs of stem cells, and thus these modules should also exist in real GRNs that maintain the differentiation processes of stem cells. Recent progress in experimental techniques such as microarray, ChIP-chip, and ChIP-Seq analysis has provided an abundant amount of information on the GRNs within stem cells and has partially identified the complex regulatory networks responsible for stem cell differentiation [35]–[40]. We expect that possible oscillation/switching modules can be screened from such putative regulatory networks. For instance, it is well known that there is a core GRN that maintains the pluripotency of ES cells, which consists of a regulatory loop comprised of Oct3/4, Nanog, and Sox2 [2]. This regulatory loop of the core network includes both positive and negative regulations [41], and thus it might correspond to our oscillation module that generates sustained oscillation ranging between high and low expression levels. In fact, we made preliminary simulations of the gene expression dynamics by using this core GRN from ES cell, and confirmed the oscillatory expression by taking appropriate parameters. Furthermore, it is established that the expression of the core network components represses the expression of lineage-specific genes [36], [38], and that these genes might also repress the expression of genes in the core GRN. Such mutual repression of the core GRN and the lineage-specific genes might be a component of the switching modules necessary for stabilizing the differentiated states through cell-cell interactions.

#### (4) Candidates of transcription factors

Several examples of ‘developmental transcription factors’ [42] are known that mutually regulate one another's expression. Transcription factors that may be responsible for stemness are systematically analyzed [43]. Importance of positive feedback loops to switching behavior of cellular states was recently observed in a response of ES cell against leukemia inhibitor factor (LIF) that controls the self-renewal of ES cells [44]. This control of switching behavior by a positive-feedback loop agrees well with our theory.

Another example of a GRN governing stem cell differentiation includes Notch signaling pathway in neural differentiation. In the cell fate switch of neural stem cell, the Notch effector Hes1 plays an essential role, whose expression is up-regulated by the activation of Notch signaling [45]. Hes1 is a bHLH transcriptional regulator, which represses the proneural gene Neurogenin-2 (Ngn2), and the expression of Ngn2 induces expression of Notch ligand as Deltalike1 (Dll1). Since the expression of Dll1 activates the Notch signaling pathway in neighboring cells, these factors, i.e., Notch/Dll1, Hes1, Ngn2, form a regulatory loop including both positive and negative regulatory interactions. This may provide an example of the oscillation module in our results. In fact, it was experimentally demonstrated that the expression levels of these factors show oscillation with the period of 2–3 hours in neural progenitors, with the aid of negative auto-regulation of Hes1 [46]. Indeed, the oscillatory expression dynamics in such regulatory loop is consistent with our theoretical prediction.

#### (5) Regulation of differentiation frequency

Robustness in the developmental process is a marvelous phenomenon, especially considering its complexity. In addition to the stability of each cell type, the number ratio of each cell type should also be robust to perturbations. Indeed, developmental processes of multicellular organisms are often “regulative,” which means that the number ratio of stem to differentiated cell types is robust with respect to perturbations. For example, in the case of mouse embryos, the removal of blastomeres from early embryos and the addition of pluripotent stem cells into the preimplantation embryos has been shown to result in normal development, which suggests that the differentiation frequencies are regulated through cell-cell interactions [47]. Regulation for maintaining the number distribution of cell types is also known to occur both in the hematopoietic system via the differentiation and proliferation of hematopoietic stem cells [48], and in the neural stem system [49].

The result here supports the previous proposition [17] that cell-cell interaction on stochastic differentiation by chaotic dynamics leads to the control of probability for proliferation of stem cells and thus leads to autonomous regulation of the number ratio of each type of cells. This type of regulation was recently observed in neural and hematopoietic stem cell systems mediated by certain factors, and models with negative-feedback loop(s) for cell-cell interaction are proposed [50]–[52]. Our interaction-based dynamic differentiation hypothesis for the regulation of differentiation frequency and robust developmental processes is consistent with these recent findings, and thus might be further confirmed experimentally in the future.

## Methods

### Gene/Protein expression dynamics and cell-cell interaction

We considered a cell whose state is represented by the expression levels of *k* genes/proteins, where the expression levels of the *i*-th mRNA and protein in the *l*-th cell at time *t* are represented as 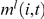 and 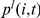, respectively. Now, all mRNA expression is regulated by some of the proteins among 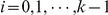. These mutual regulations constitute a GRN. Proteins activate, inhibit, or fail to influence the expression of each gene. As a simple model, we assumed that the change of the *i*-th mRNA expression level is given by
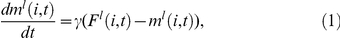



with



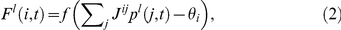



where 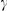, which is the rate of the change in mRNA, was set to 6 [53]–[55]. Here, the function 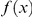 approaches 1, as *x* is increased with a positive value, and approaches 0 as *x* is decreased with a negative value. In other words, if 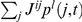 is larger than the threshold 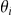, then 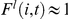, which indicates that the gene is fully expressed, and if it is smaller than the threshold, then 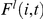 goes to zero, which indicates that the gene expression is suppressed. Here, we chose, 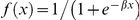, where 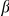, which was set to 40, denotes the slope of the threshold. Roughly speaking, larger values of 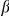 correspond to a larger Hill coefficient. The matrix
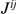
represents the GRN: 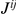 is 1, if the protein *j* activates the expression of the gene *i*, it is −1 if it suppresses the expression, and it is 0 if there is no connection. The term 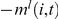 represents the degradation of the mRNA. Here, we normalized the maximum (saturated) concentration of the mRNA as 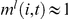.

Next, the protein was synthesized from each mRNA. By again normalizing the maximal concentration as 1, the dynamics of protein concentration are given by
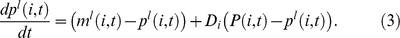



Here, the first 2 terms represent the synthesis of the protein from the mRNA and its degradation, respectively. The time-scale for protein degradation is unity, which is larger than that for mRNA (Eq. (1)), which is *1/* 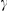
( = 1/6). The term with 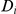 shows the diffusion of a penetrable protein, where 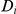 is the diffusion constant is set to 0.4, unless otherwise mentioned. For non-penetrable proteins, it is instead set to 0. For simplicity, we considered a well-mixed liquid culture; 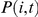 represents the concentration of the protein in this medium. Assuming a fast global diffusion from all cells, 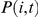 is given by the average of the protein concentration over all cells, i.e., 
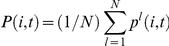
.

We started the simulation of this model from a single cell whose initial expression level was determined randomly. Then, at a given time, we assumed it divided into 2 cells. Upon division, the concentration of each protein (and mRNA) was almost entirely preserved for the divided cells, and was only slightly perturbed. Here, we took a small random number 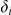 over [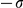,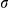], where 1 cell that arose from the mother cell 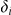 was added to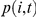, and for the other cell it was subtracted. This “noise” level was set to 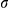, which was set to 0.00001, unless otherwise indicated. The cell division was repeated 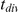 after its last division, such that the cell number increased as 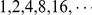 per 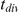, which was set to 500, unless otherwise indicated. The threshold 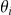 was set to nearly 0, although we slightly changed its value for proteins to eliminate artificial symmetric solution over different proteins. We repeated the cell division up to *N = 128.*

For enumerative simulation, we studied all possible networks with 5 genes (*k* = 5) and 10 paths (i.e., 10 elements in the matrix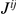, where 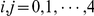, and was set to 1 or −1, and others were set to 0). We examined all possible networks. Although 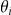 was set close to zero, it was actually set to a value slightly different than zero to eliminate artificial symmetry among all proteins. Specifically, we set 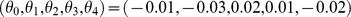. In these simulations, the parameter values of 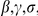 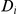, and 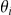 were fixed. As for the values of 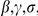 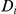, the results here are rather insensitive; The value 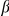 need to be sufficiently large, say larger than 25; 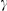 is larger than 1, while if it is much larger than 10, the frequency of oscillation behavior and accordingly the differentiation is reduced. The noise level 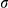 can take any values as long as it is larger than zero and not too large (say less than .3 or so). As shown in Fig. 4, even if it is of the order of 0.1, the results reported here are not changed. If 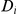 is of the order of 0.1, we observe the differentiation. The threshold values 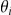 are more important. The differentiation is preserved within the change of 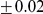 or so, but by varying these parameters of the order of .1, differentiation could be lost for each network. Still, one might find other examples of GRNs with differentiations, as long as 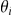
's are close to zero.

### Procedure to select networks allowing differentiation

When the cell number was 32, we determined whether there were distinct cell types. Here, we computed the average protein concentration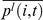 over the time-span 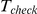, which was set to 100. We subsequently computed the Euclidean distance 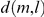 in the protein state-space 
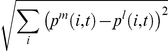
 between the *m*-th cell and the *l*-th cell. If the distance of at least one of the pairs of *m* and *l* was larger than a given threshold 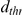
(set at 0.3), we considered that there were different cell types. For differentiated cell types, some of proteins are either expressed to maximal or minimal levels, i.e., 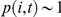 or ∼0, so that the distance between distinct cell types is typically larger than 0.5, and hence this choice of 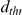 is sufficient. If 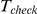
could be chosen arbitrarily large, the distinction of types by arbitrarily small 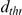
value could be accurate, as the averaging out of oscillation would be perfect. On the other hand, if 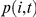 showed a temporal change larger than the scale 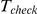, then a long-term change in a single cellular state was regarded as differentiation. Here, we set 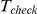 sufficiently large ( = 100) to reduce such miscounting. For this case, by taking 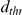 = *(0.1*∼*0.4)*, there were only slight changes in the result. Still, there remained a few cases that were eliminated by directly checking the time-series.

### Procedure for selecting networks with stem cells

Since we sought a system with a stem cell, it was necessary for one type of cells to both proliferate and differentiate repeatedly. To further select such networks we applied the following procedure against all the above selected networks with regards to just differentiation. We simulated the gene/protein expression dynamics in the presence of cell-cell interactions and with an increase in cell number up to 256. We subsequently checked whether plural differentiation events occurred from one type to another, and selected such case. In other words, from the GRNs showing plural differentiations, we discarded the case in which differentiation from one type 1 to another type 2 occurred first, and then from type 2 to type 1 in a later generation. Since the average concentrations of each cell type could vary with the increase in cell number, identification of the same cell type might be sometimes difficult algorithmically. There were three ‘gray’ cases, which were removed here, and we finally selected 231 cases. (Among them there were about 20 subtle cases in which the protein concentrations change in a long-time scale, in which 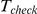 to obtain the temporal average might not be yet sufficiently long.)

## Supporting Information

Figure S1 **List of all networks that showed differentiation repeatedly, which included 231 networks.** The first 8 networks were reduced to 4 networks of 3 genes, and the next 2 panels of a total of 79 networks was reduced to 65 networks of 4 gene networks (see the main text). The next 4 panels of a total of 146 networks were inherently five-gene networks.
(PDF)

Figure S2
**Example of differentiation only of the phase of oscillations, but not of the differentiations.** The trajectories 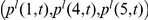 are plotted over 8 cells. The values of each cell at 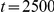 are plotted as circles of different colors. Even though the cellular states were identical, the protein concentrations at each snapshot differed among cells, as the phases of oscillation were scattered following chaotic oscillation. However, the protein level did not show switch between “on” and “off” states.(PDF)

Figure S3
**Change in the flow in protein state space.** By taking an example in Fig. 1(c), plotted were trajectories of 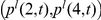 of a “test cell” that is influenced only from 32 cells of the original model with the network in Fig. 1(c1), whereas it did not influence other cells. Trajectories from.25 initial conditions within the dotted ellipse are displayed with different colors. (a) The expression dynamics were computed under the presence of other cells developed in the same way as in Fig. 1(c2) for given time span. (a1) *0<t<4*, when there was no other cell. All the trajectories were attracted to the original attractor. (a2) *2650<t<2654*, under the presence of 32 cells developed from a single cell as in Fig. 1(c). Among 25 initial states of the test cell in the figure, 21 were attracted to the original attractor (flows to the left), while 4 were attracted to a new state (flows to the right), corresponding to a differentiated cell type. Indeed cell differentiation event occurred around this time step. (a3) *2679<t<2683,* right after the event of cell differentiation (see Fig. 1(c2)), under the presence of 32 cells. At this stage all the 25 initial conditions in the figure were attracted into the original attractor. (b) The expression dynamics of a test cell were plotted for 4 time units, under the presence of 32 cells of the two cell types whose numbers were preset as the original cell type at 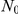, and that of the differentiated type at 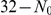. (b1) 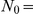 8, (b2) 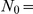 12, (b3) 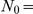 16, and (b4) 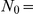 32. When 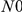 was less than or equal to 8, all the initial conditions were attracted to the original cell type (attractor, left in the figure), as shown in (b1). As the number 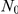 was increased, some initial conditions (cell states) were attracted to the differentiated cell type (right in the figure). The fraction of initial conditions attracted to this differentiated type increased as 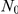 was increased.(PDF)

Figure S4
**State differentiation represented by orbits in the state-space of 3 protein concentrations.** (a) 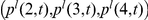 is plotted for 7 cells 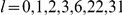, over 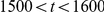, for the gene regulatory network of the fourth item in the panel of Fig. S1, which is reduced to the network shown in Fig. 5(d). Differentiation from the original attractor (left) to a new state (right) progressed. (b) The time-series of the protein concentration 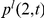 is plotted. The differentiations occurred at around *t* = 550, 1100, 1600, and 2600.(PDF)

Figure S5 **Designed gene regulatory network to produce differentiation stem cell (S)→A,B/A→A_1_/A→A_2_/A_2_→A_3_/A_3_→A_4_/B→B_1_.**
(PDF)

Movie S1The temporal change of 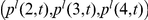 plotted for 7 cells, shown through the time up to *t  = 1200*, for the gene regulatory network shown in Fig. 1(c). Different colors represent different cells. In this movie, 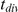 was set to 200, instead of 500, to allow observation of several differentiations within a shorter time span.(MPG)

Movie S2The temporal change of 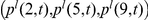 plotted for 6 cells, shown through the time up to *t  = 960*, for the gene regulatory network in Fig. 7(a). Different colors represent different cells. In this movie, 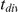 was set to 160, instead of 500, to allow observation of several differentiations within a shorter time span. In the following three movies the diffusion constant was set to 0.125.(MPG)

Movie S3The temporal change of 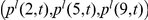 plotted for 6 cells, shown through the time up to *t = 960*, for the gene regulatory network in Fig. 7(b). Different colors represent different cells. In this movie 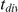
is set at 160, instead of 500, to allow observation of several differentiations within a shorter time span.(MPG)

Movie S4The temporal change of 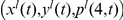 plotted for 14 cells, shown through the time up to *t  = 1264*, for the gene regulatory network in Fig. S5. Different colors represent different cells. To distinguish the 7 cell types, we adopted the coordinates,
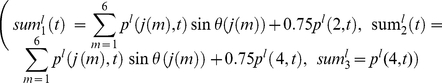
where genes 0,1, 2, 3, and 4, are motifs of the chaotic oscillations, 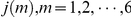 assigns each gene that is expressed for each of the 6 types (A, A1, A2, A3, B, and B1) specifically, and 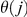 is defined as 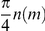 with 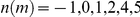
for 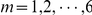, which are adopted to clearly distinguish the 7 types in the plot (but have no meaning). In this movie, 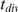 was set to 158, instead of 500, to allow observation of several differentiations within a shorter time span.(MPG)
