# Supplementary material for: Oscillatory Protein Expression Dynamics Endows Stem Cells with Robust Differentiation Potential
Source: PLoS One. 2011 Nov 3;6(11):e27232. doi: 10.1371/journal.pone.0027232 (PMC3207845; doi:10.1371/journal.pone.0027232)
Supplement: Figure S1 — List of all networks that showed differentiation repeatedly, which included 231 networks. The first 8 networks were reduced to 4 networks of 3 genes, and the next 2 panels of a total of 79 networks was reduced to 65 networks of 4 gene networks (see the main text). The next 4 panels of a total of 146 networks were inherently five-gene networks. (PDF) [file pone.0027232.s001.pdf]

3-gene networks that showed differentiation repeatedly

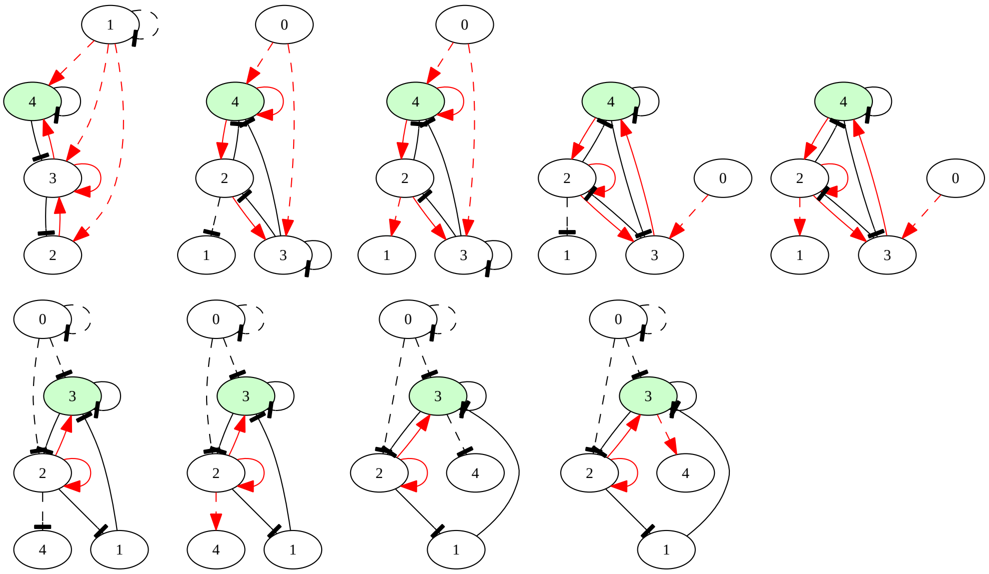

4-gene networks that showed differentiation repeatedly

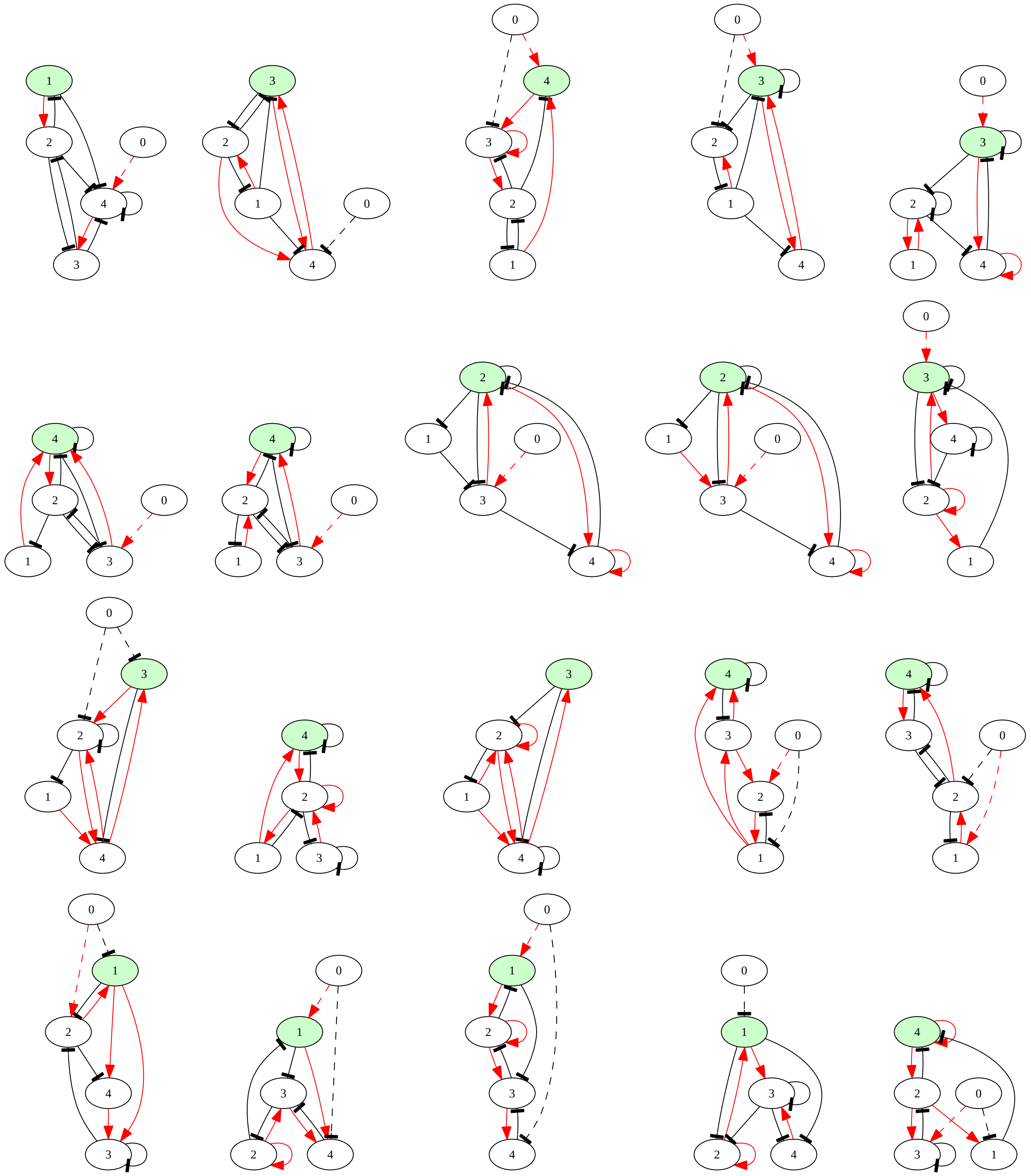

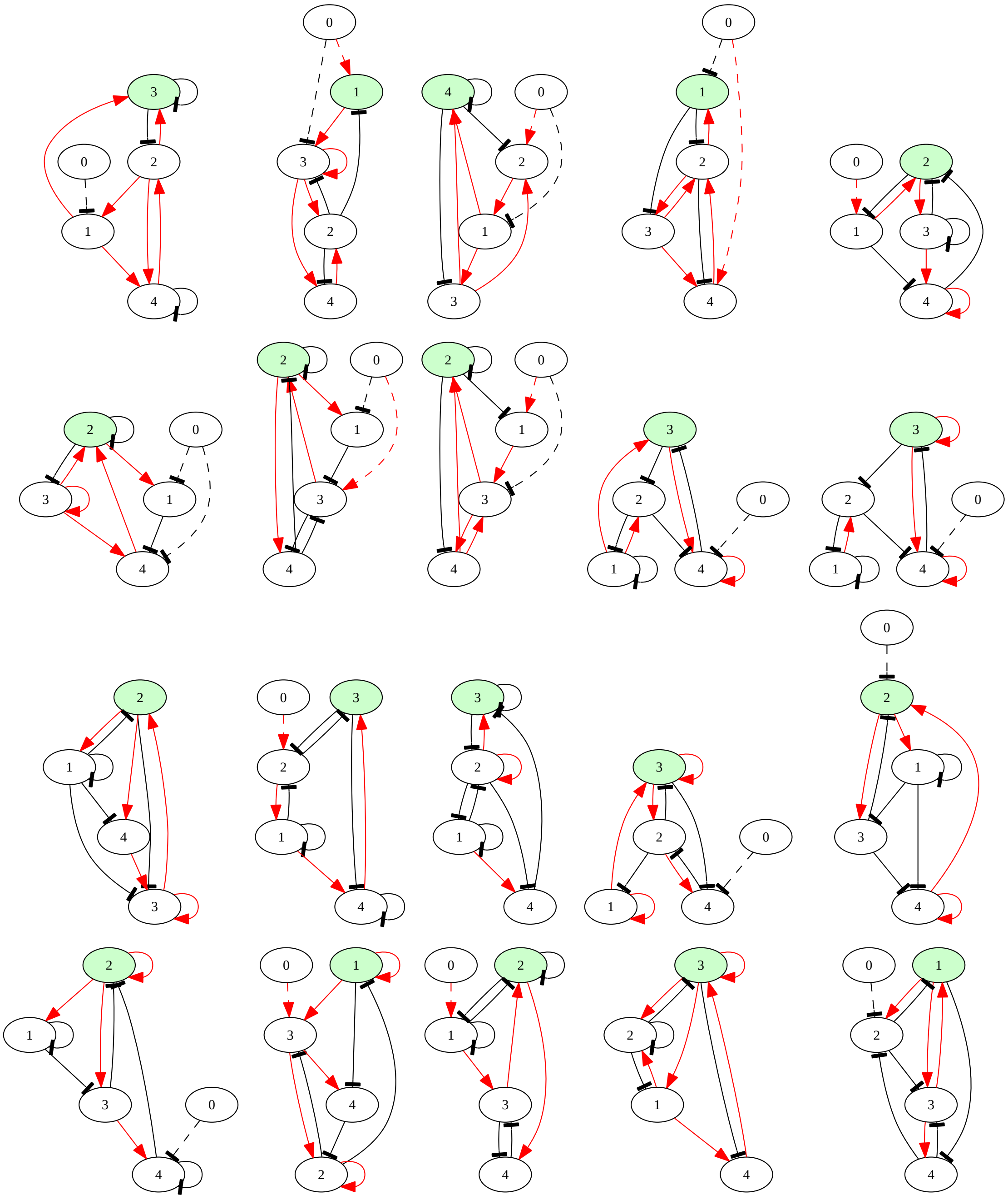

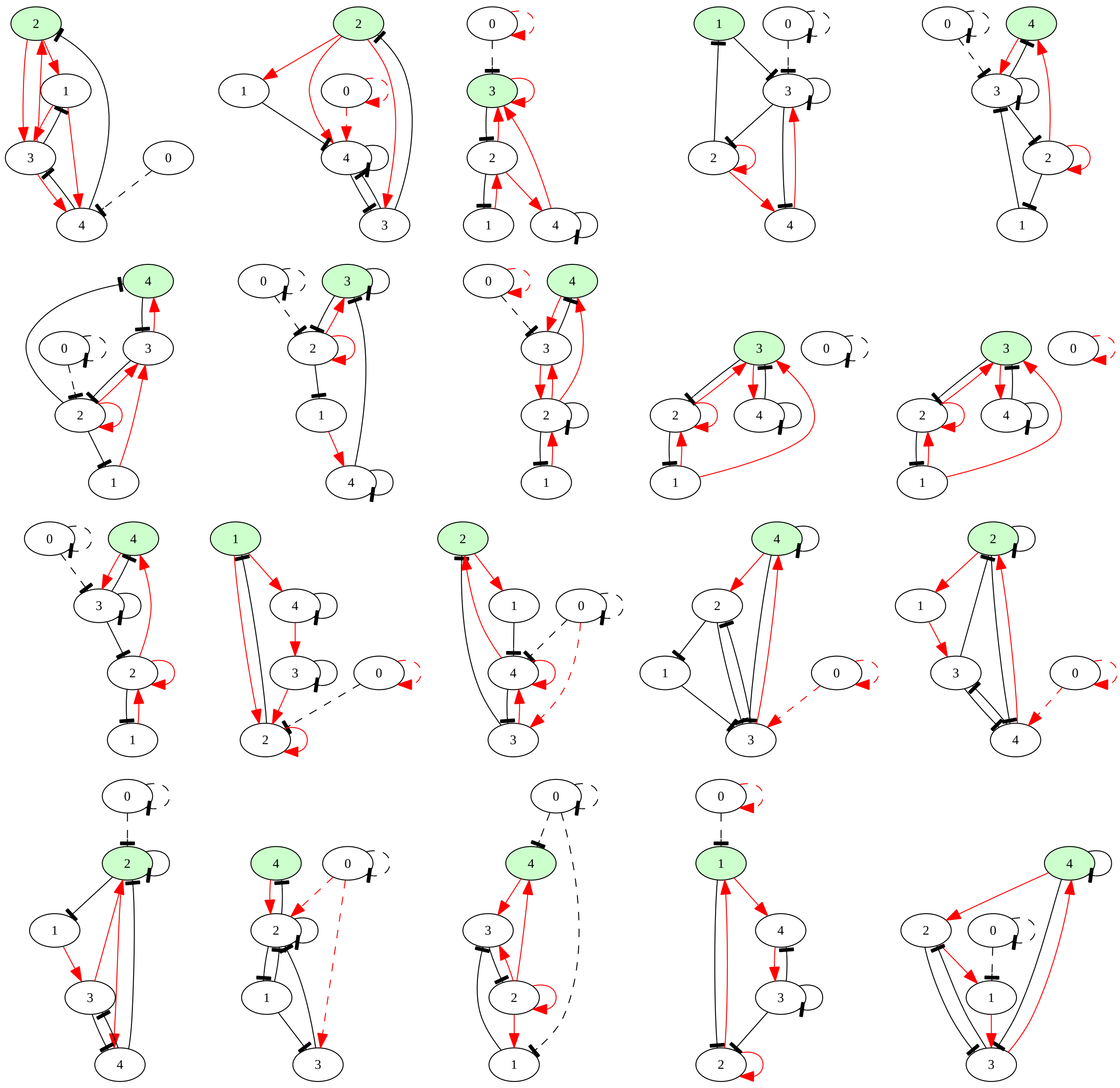

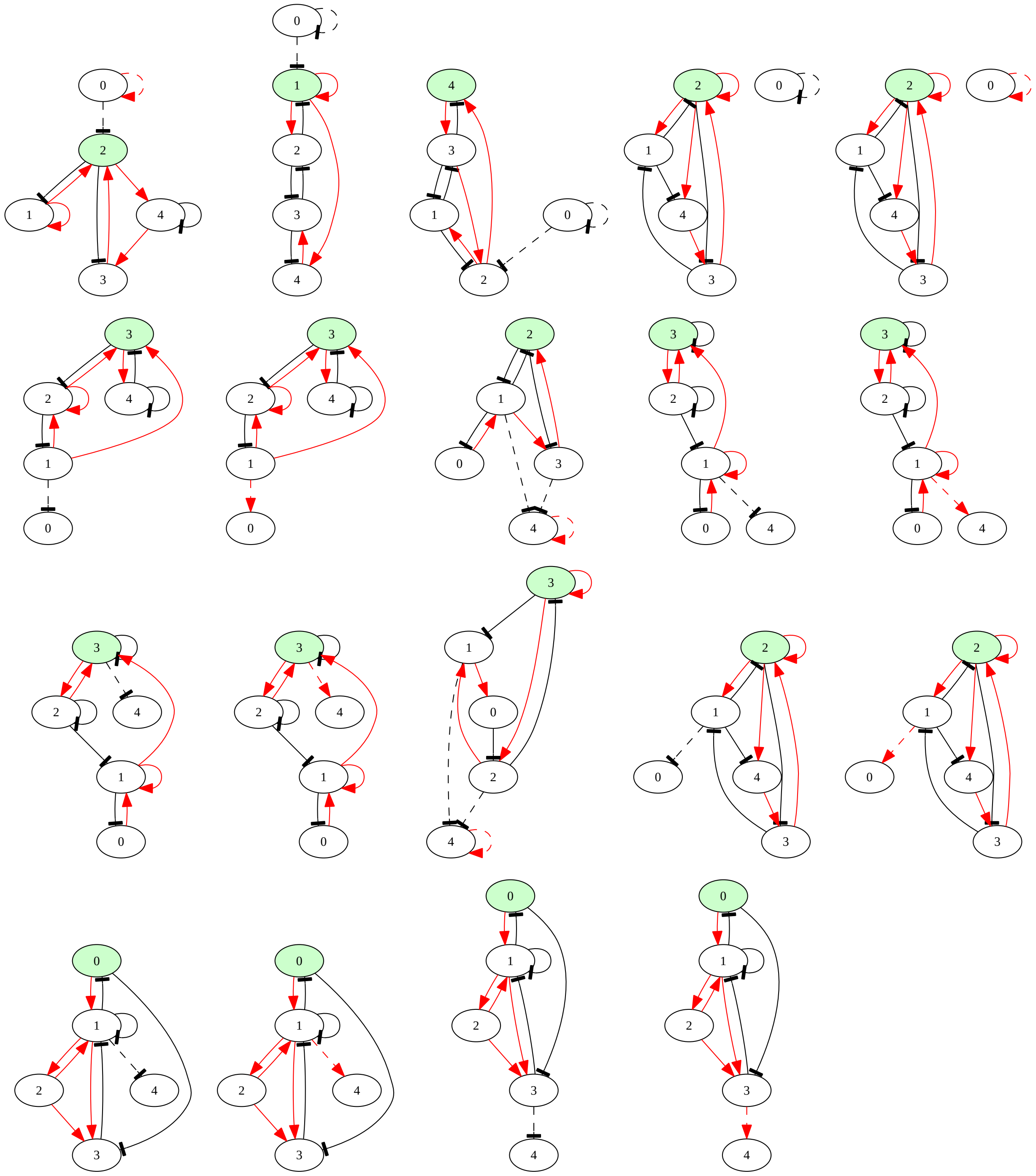

5-gene networks that showed differentiation repeatedly

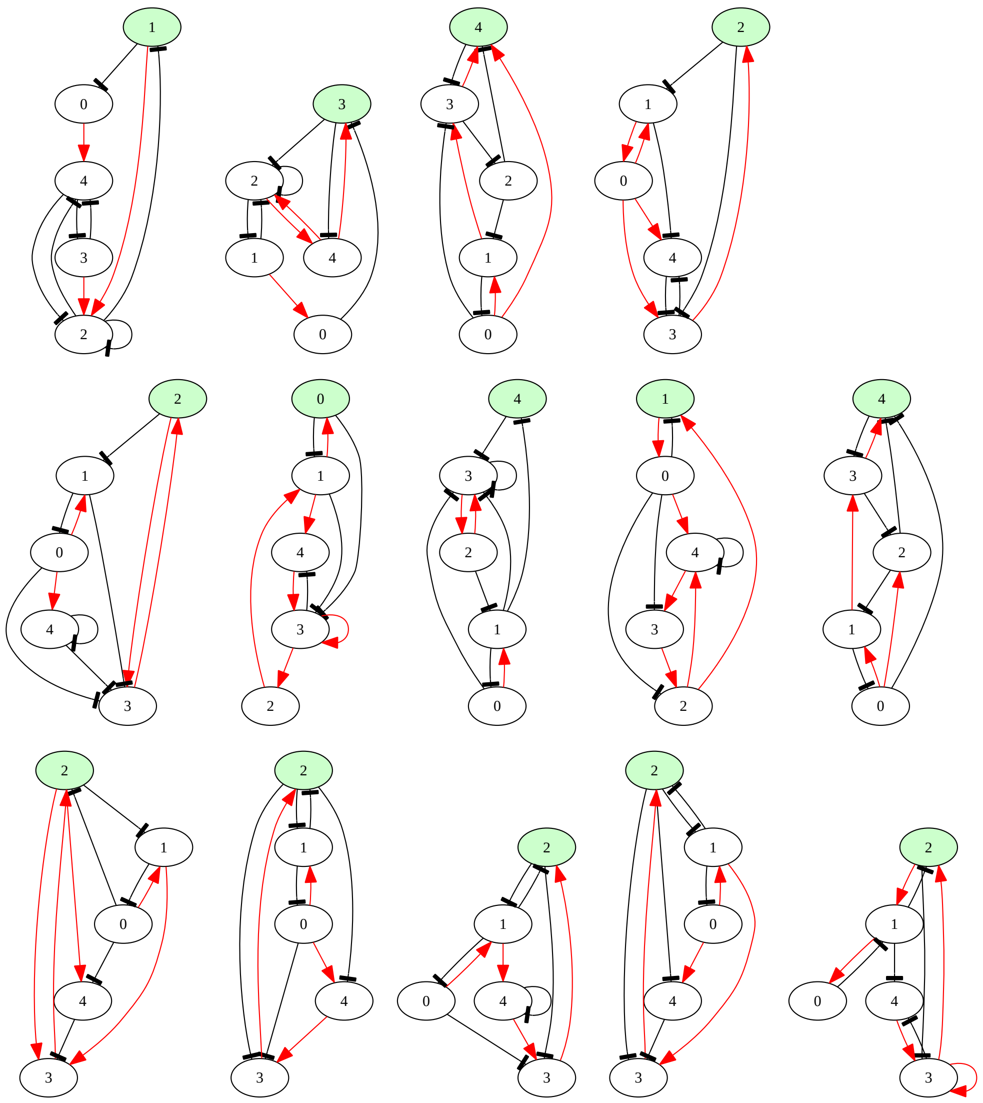

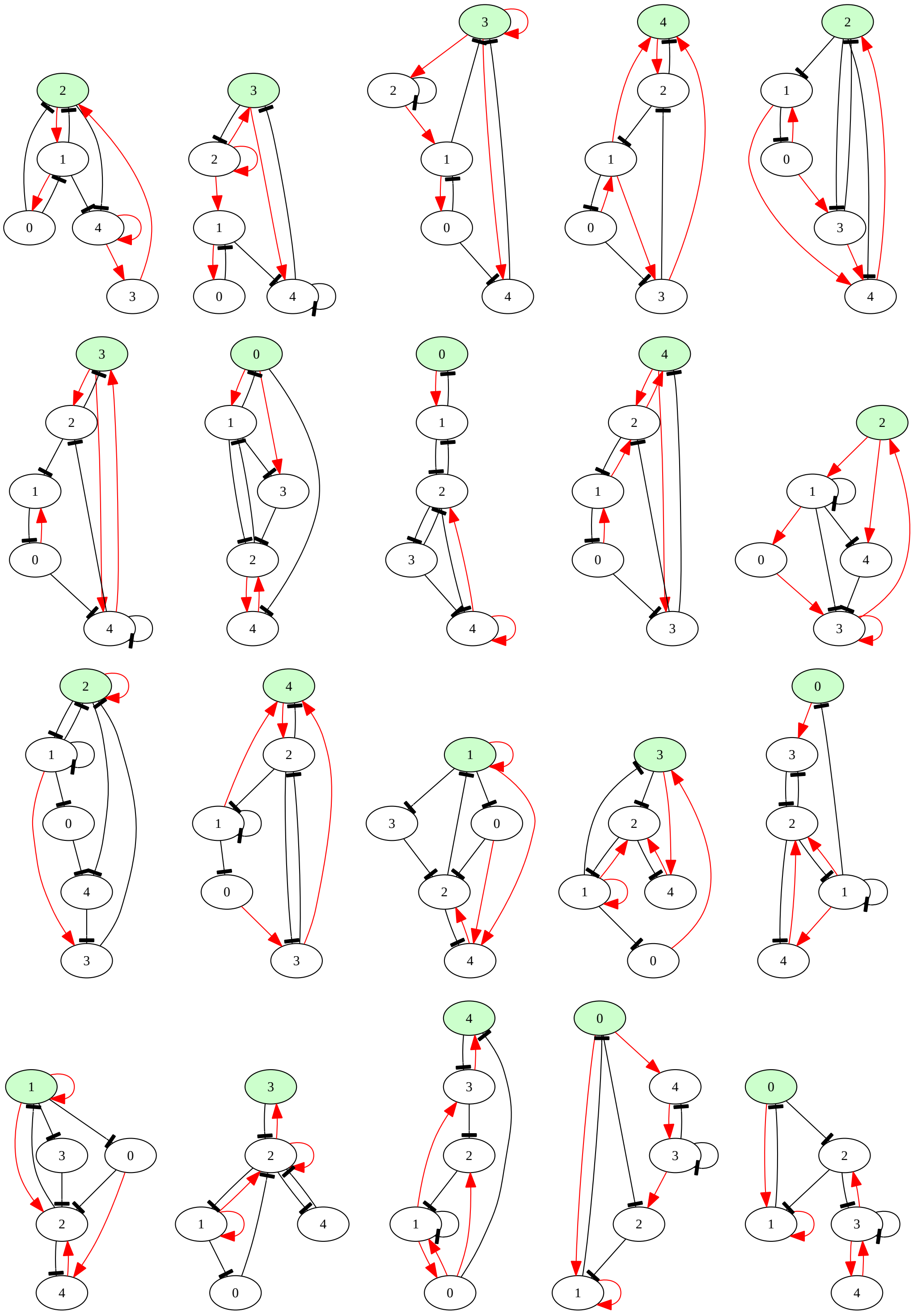

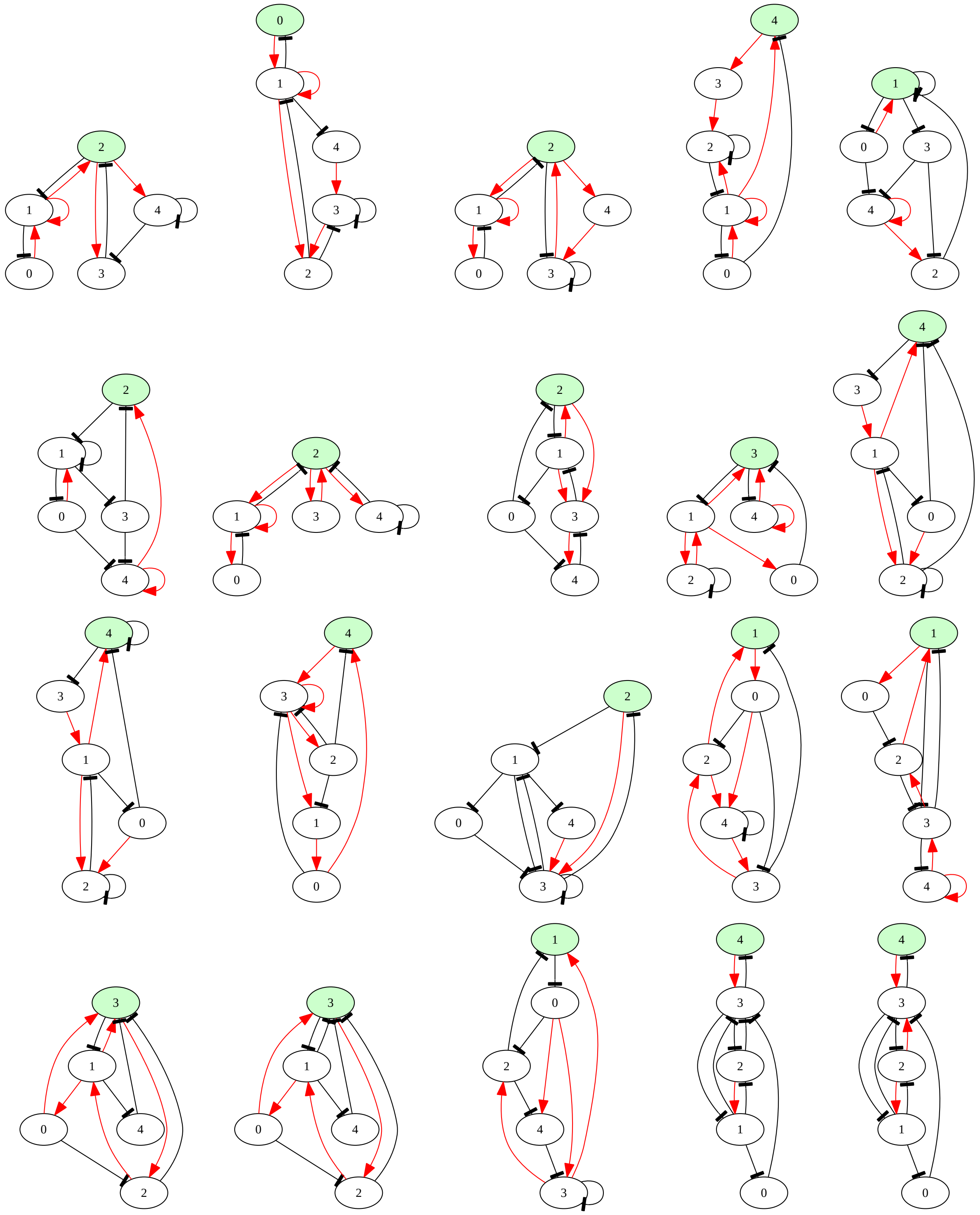

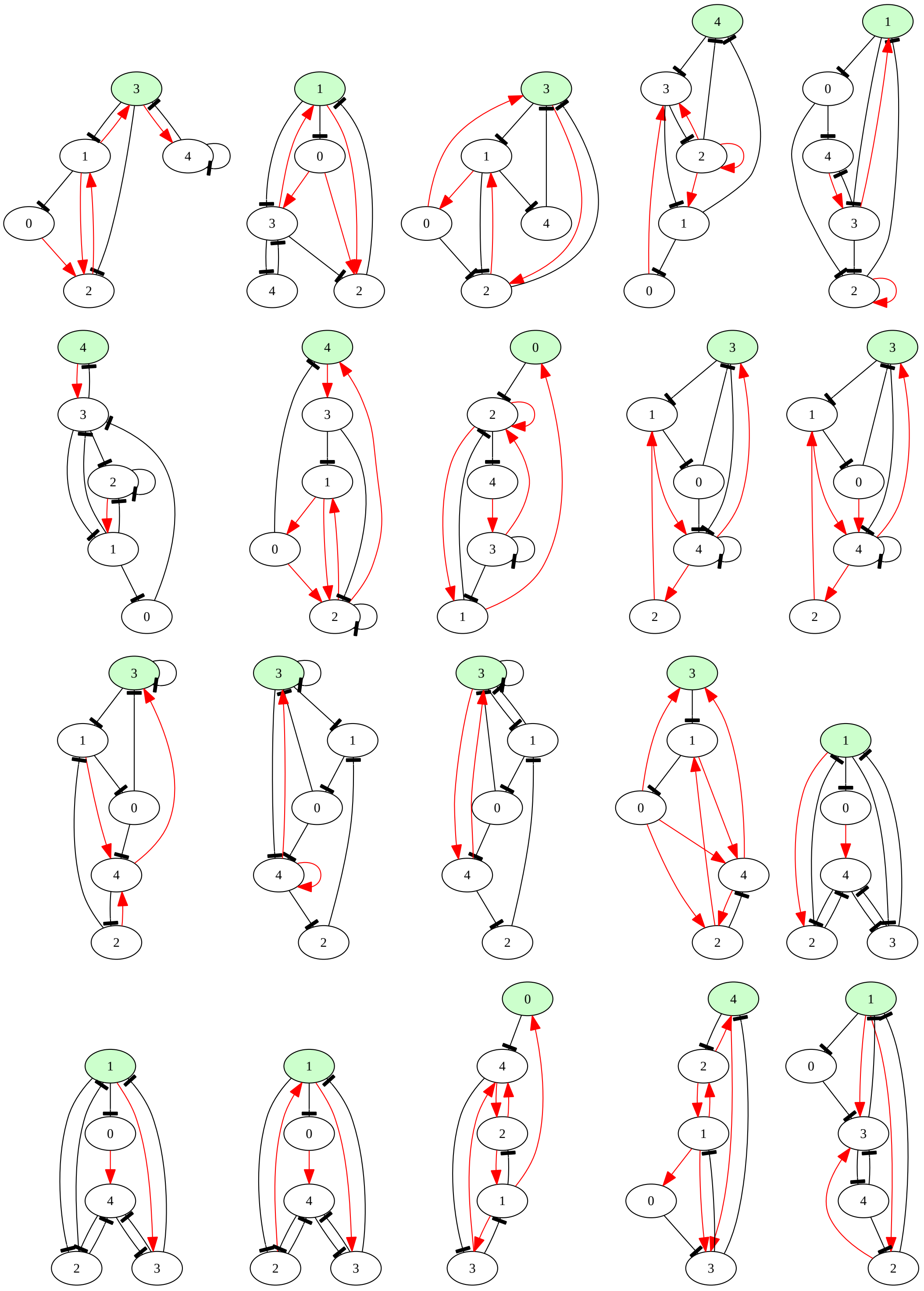

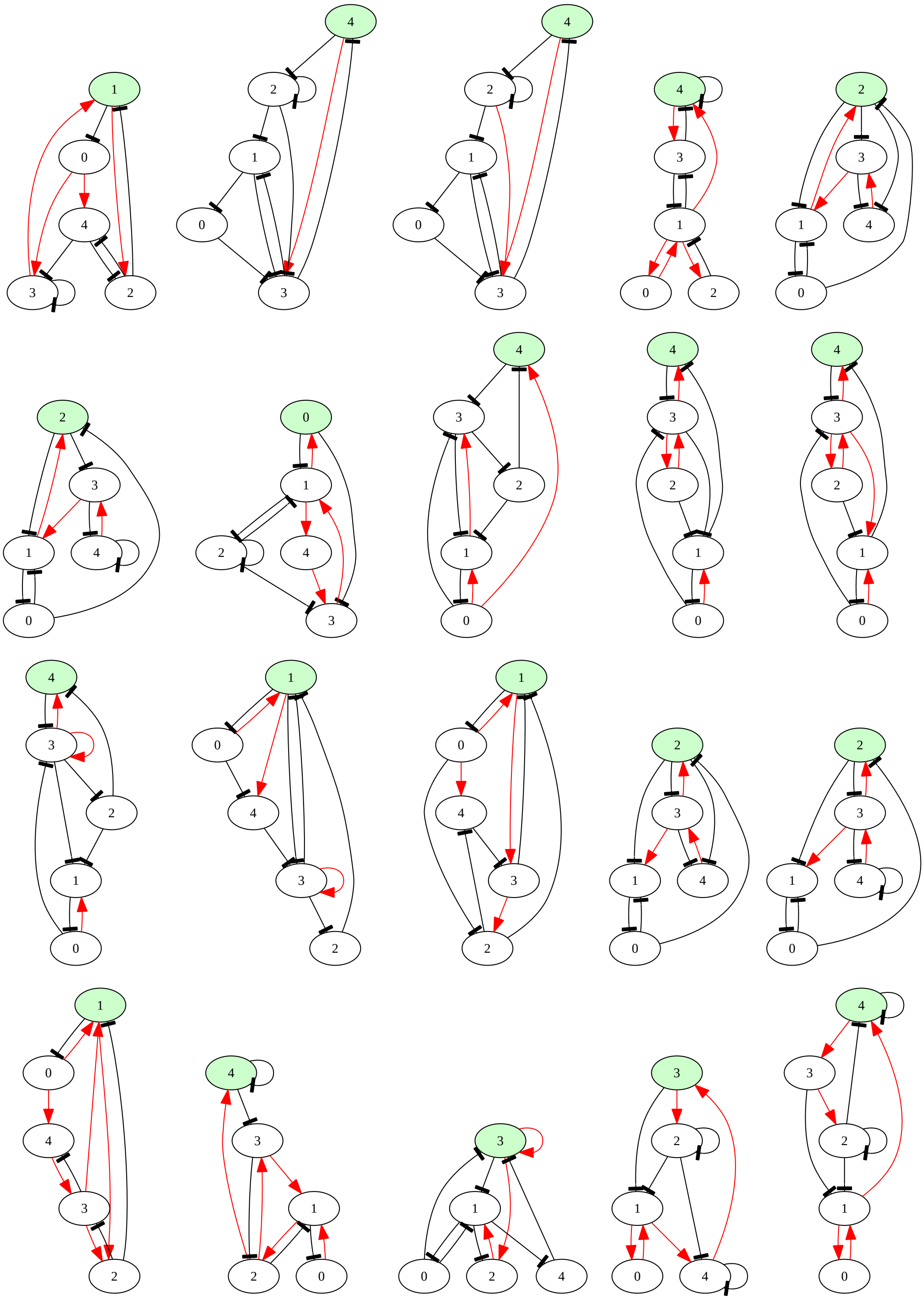

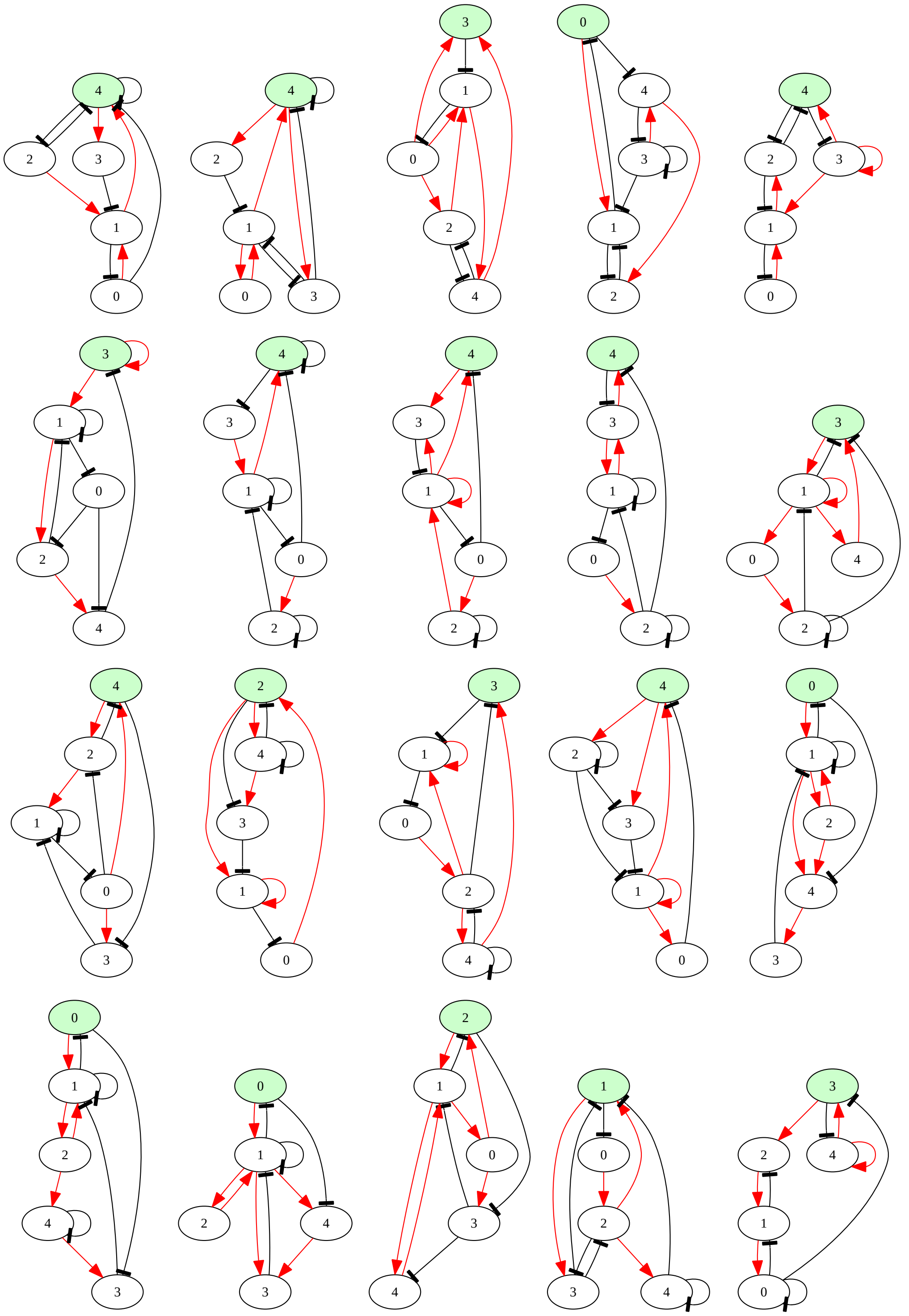

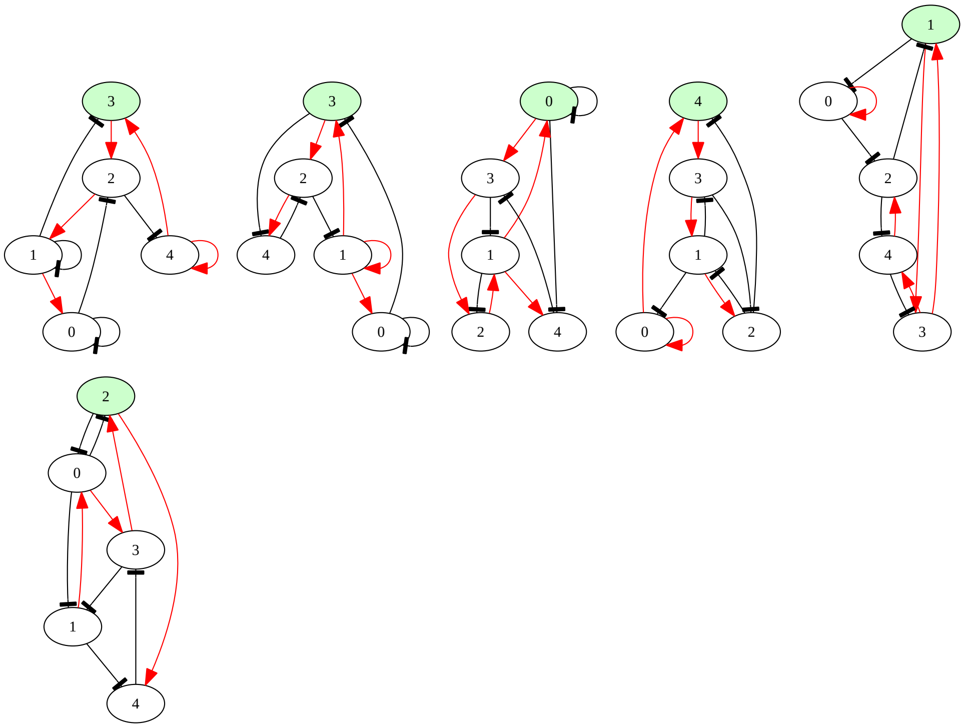
3 2 1 4 0
3 2 4 1 0
0 3 1 2 4
4 3 1 0 2
1 0 2 4 3
2 0 3 1 4
