# Supplementary figures and images for: Oscillatory Protein Expression Dynamics Endows Stem Cells with Robust Differentiation Potential
Source: PLoS One. 2011 Nov 3;6(11):e27232. doi: 10.1371/journal.pone.0027232 (PMC3207845; doi:10.1371/journal.pone.0027232)

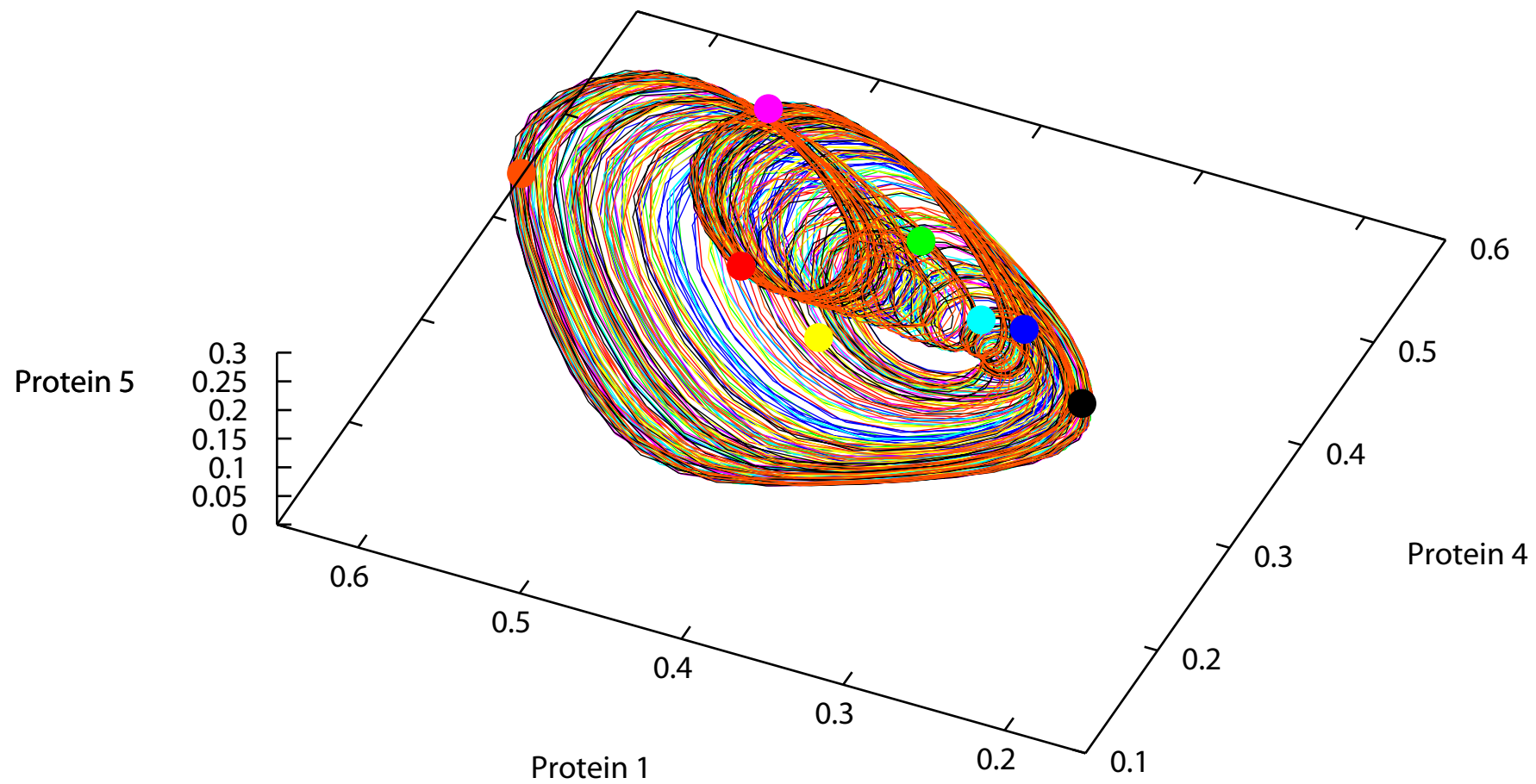

Supplement: Figure S2 — Example of differentiation only of the phase of oscillations, but not of the differentiations. The trajectories are plotted over 8 cells. The values of each cell at are plotted as circles of different colors. Even though the cellular states were identical, the protein concentrations at each snapshot differed among cells, as the phases of oscillation were scattered following chaotic oscillation. However, the protein level did not show switch between “on” and “off” states. (PDF) [file pone.0027232.s002.pdf]

(a1)

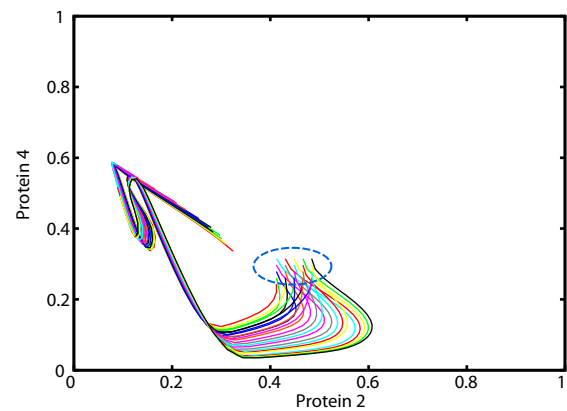

(a2)

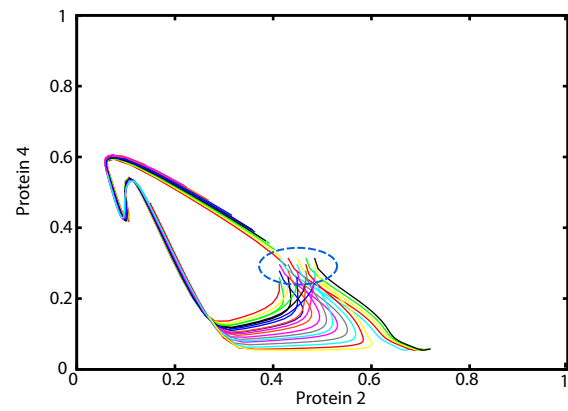

(a3)

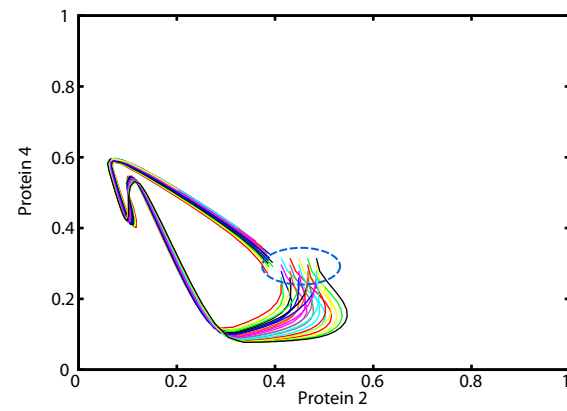

(b1)

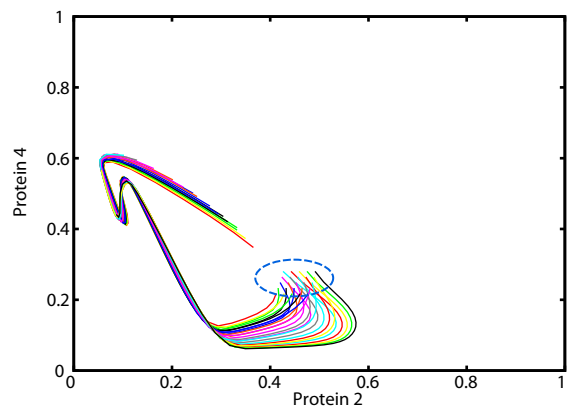

(b2)

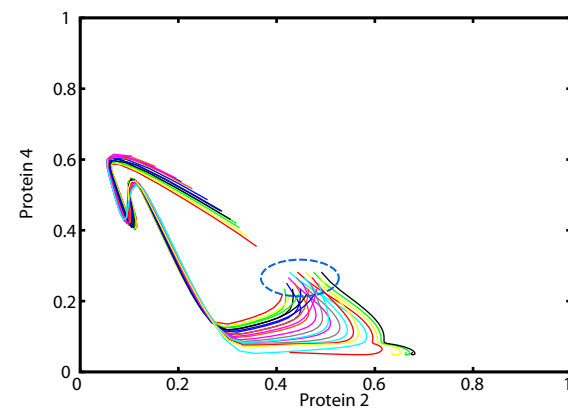

(b3)

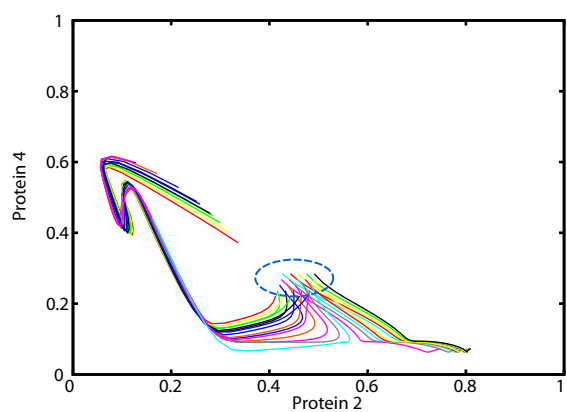

(b4)

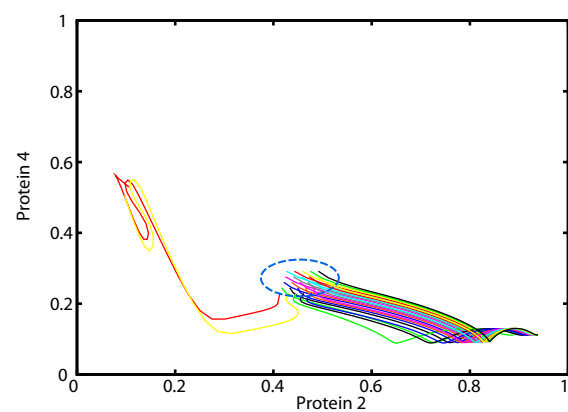

Supplement: Figure S3 — Change in the flow in protein state space. By taking an example in Fig. 1(c), plotted were trajectories of of a “test cell” that is influenced only from 32 cells of the original model with the network in Fig. 1(c1), whereas it did not influence other cells. Trajectories from.25 initial conditions within the dotted ellipse are displayed with different colors. (a) The expression dynamics were computed under the presence of other cells developed in the same way as in Fig. 1(c2) for given time span. (a1) 0<t<4, when there was no other cell. All the trajectories were attracted to the original attractor. (a2) 2650<t<2654, under the presence of 32 cells developed from a single cell as in Fig. 1(c). Among 25 initial states of the test cell in the figure, 21 were attracted to the original attractor (flows to the left), while 4 were attracted to a new state (flows to the right), corresponding to a differentiated cell type. Indeed cell differentiation event occurred around this time step. (a3) 2679<t<2683, right after the event of cell differentiation (see Fig. 1(c2)), under the presence of 32 cells. At this stage all the 25 initial conditions in the figure were attracted into the original attractor. (b) The expression dynamics of a test cell were plotted for 4 time units, under the presence of 32 cells of the two cell types whose numbers were preset as the original cell type at , and that of the differentiated type at . (b1) 8, (b2) 12, (b3) 16, and (b4) 32. When was less than or equal to 8, all the initial conditions were attracted to the original cell type (attractor, left in the figure), as shown in (b1). As the number was increased, some initial conditions (cell states) were attracted to the differentiated cell type (right in the figure). The fraction of initial conditions attracted to this differentiated type increased as was increased. (PDF) [file pone.0027232.s003.pdf]

(a)

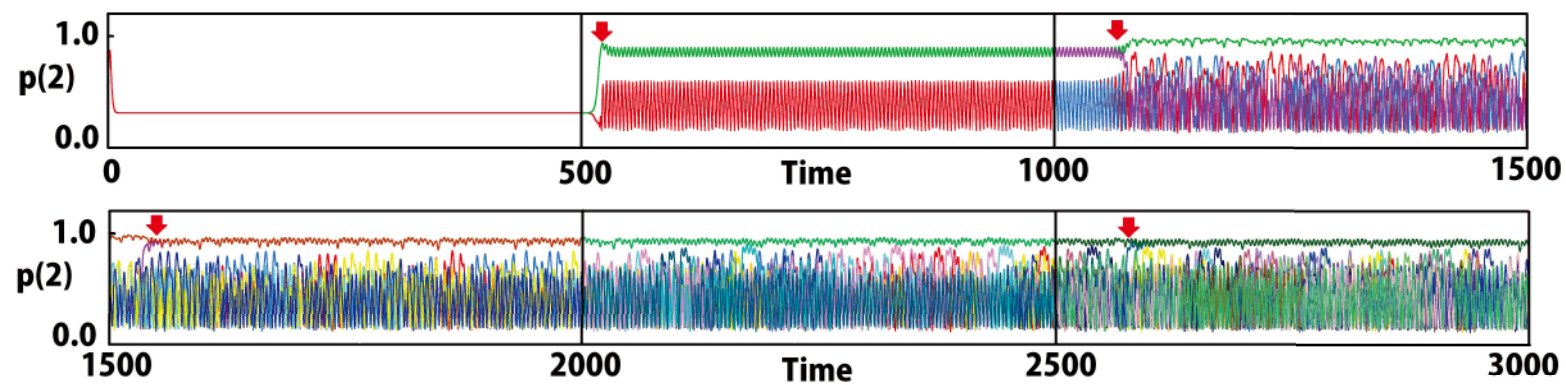

(b)

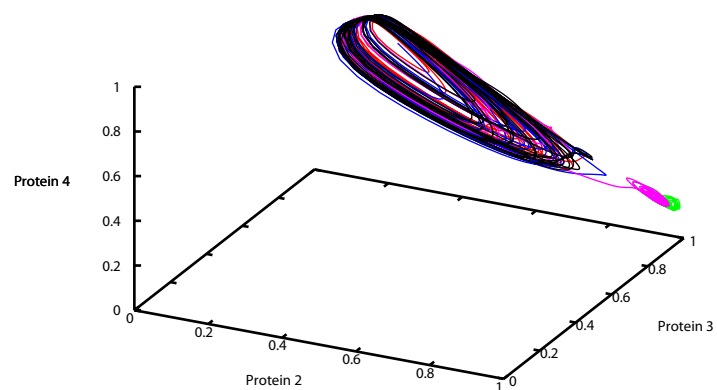

Supplement: Figure S4 — State differentiation represented by orbits in the state-space of 3 protein concentrations. (a) is plotted for 7 cells , over , for the gene regulatory network of the fourth item in the panel of Fig. S1, which is reduced to the network shown in Fig. 5(d). Differentiation from the original attractor (left) to a new state (right) progressed. (b) The time-series of the protein concentration is plotted. The differentiations occurred at around t = 550, 1100, 1600, and 2600. (PDF) [file pone.0027232.s004.pdf]

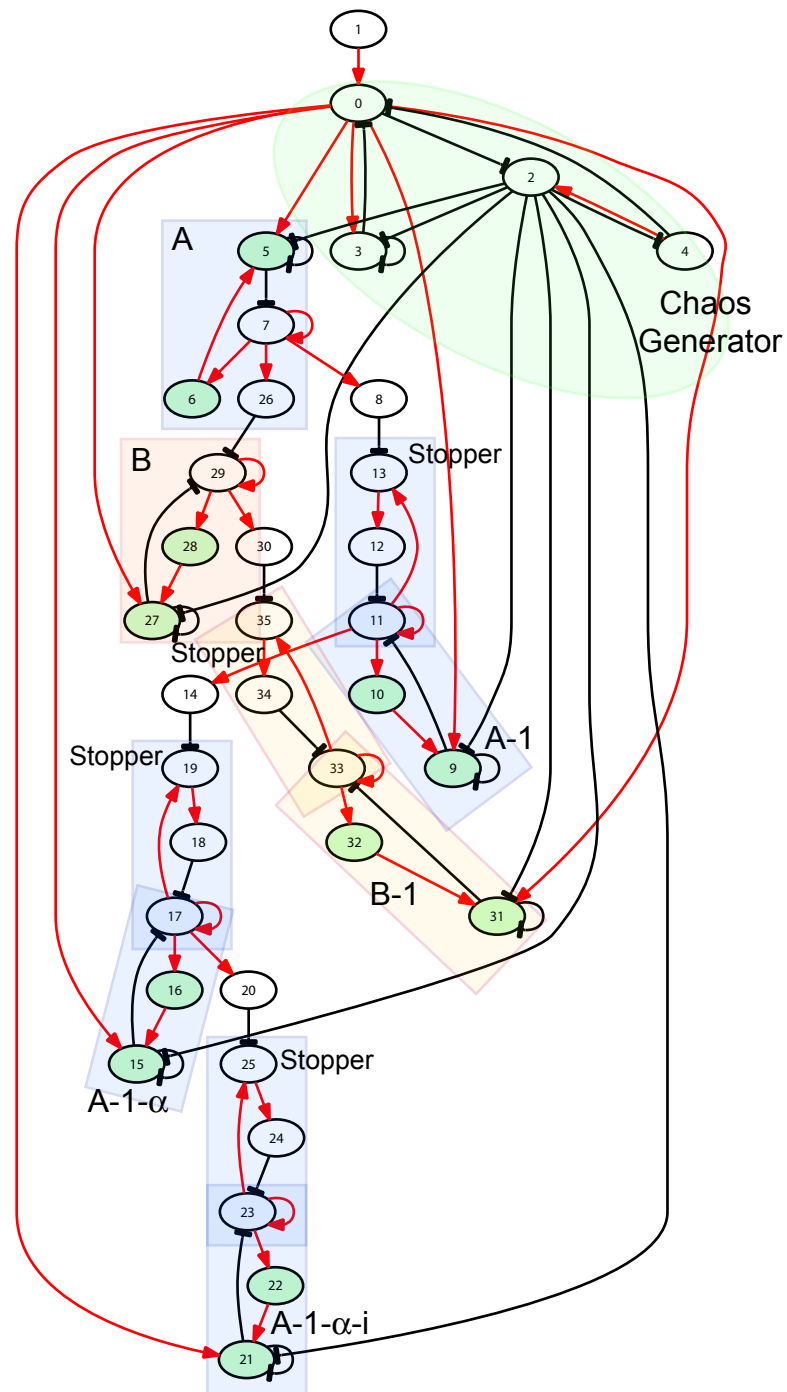

Supplement: Figure S5 — Designed gene regulatory network to produce differentiation stem cell (S)→A,B/A→A1/A→A2/A2→A3/A3→A4/B→B1. (PDF) [file pone.0027232.s005.pdf]
